# Plasma proteome plus site‐specific *N*‐glycoprofiling for hepatobiliary carcinomas

**DOI:** 10.1002/cjp2.136

**Published:** 2019-06-25

**Authors:** Ting‐Tsung Chang, Ji‐Hong Cheng, Hung‐Wen Tsai, Kung‐Chia Young, Sun‐Yuan Hsieh, Cheng‐Hsun Ho

**Affiliations:** ^1^ Department of Internal Medicine National Cheng Kung University Hospital, College of Medicine, National Cheng Kung University Tainan Taiwan; ^2^ Institute of Molecular Medicine, College of Medicine, National Cheng Kung University Tainan Taiwan; ^3^ Department of Computer Science and Information Engineering College of Electrical Engineering and Computer Science, National Cheng Kung University Tainan Taiwan; ^4^ Department of Pathology National Cheng Kung University Hospital, College of Medicine, National Cheng Kung University Tainan Taiwan; ^5^ Department of Medical Laboratory Science and Biotechnology College of Medicine, National Cheng Kung University Tainan Taiwan; ^6^ Department of Medical Laboratory Science College of Medicine, I‐Shou University Kaohsiung City Taiwan

**Keywords:** hepatocellular carcinoma, cholangiocarcinoma, proteomic, glycosylation, mass spectrometry

## Abstract

Hepatobiliary cancer is the third leading cause of cancer death worldwide. Appropriate markers for early diagnosis, monitoring of disease progression, and prediction of postsurgical outcome are still lacking. As the majority of circulating *N*‐glycoproteins are originated from the hepatobiliary system, we sought to explore new markers by assessing the dynamics of *N*‐glycoproteome in plasma samples from patients with hepatocellular carcinoma (HCC), cholangiocarcinoma (CCA), or combined HCC and CCA (cHCC‐CCA). Using a mass spectrometry‐based quantitative proteomic approach, we found that 57 of 5358 identified plasma proteins were differentially expressed in hepatobiliary cancers. The levels of four essential proteins, including complement C3 and apolipoprotein C‐III in HCC, galectin‐3‐binding protein in CCA, and 72 kDa inositol polyphosphate 5‐phosphatase in cHCC‐CCA, were highly correlated with tumor stage, tumor grade, recurrence‐free survival, and overall survival. Postproteomic site‐specific *N*‐glycan analyses showed that human complement C3 bears high‐mannose and hybrid glycoforms rather than complex glycoforms at Asn85. The abundance of complement C3 with mannose‐5 or mannose‐6 glycoform at Asn85 was associated with HCC tumor grade. Furthermore, stepwise Cox regression analyses revealed that HCC patients with a hybrid glycoform at Asn85 of complement C3 had a lower postsurgery tumor recurrence rate or mortality rate than those with a low amount of complement C3 protein. In conclusion, our data show that particular plasma *N*‐glycoproteins with specific *N*‐glycan compositions could be potential noninvasive markers to evaluate oncological status and prognosis of hepatobiliary cancers.

## Introduction

Hepatobiliary cancer ranks sixth in the world among all malignancies and is the third leading cause of cancer mortality. Hepatocellular carcinoma (HCC) is the most common primary hepatic malignancy, with an average survival period between 6 and 20 months 1. Risk factors for HCC include chronic hepatitis B or C virus infection, alcoholic liver disease, steatohepatitis, and liver cirrhosis. Cholangiocarcinoma (CCA), appearing as an intrahepatic type, a perihilar type (also known as Klatskin tumor), or a distal extrahepatic type, is the second most common liver cancer 2. In contrast to the high prevalence of HCC (more than 700 000 new cases diagnosed every year globally), CCA has an annual incidence rate of approximately 2 per 100 000 people in western countries and 5 per 100 000 people in northeastern Asia 3, 4, 5. Nevertheless, the overall incidence of CAA has increased over the past four decades. Risk factors for CCA include primary sclerosing cholangitis, liver fluke infection (*Opisthorchis viverrini*), chronic ulcerative colitis, biliary malformation (choledochal cysts or Caroli's disease), and thorotrast 6.

Diagnosing hepatobiliary cancers at an early stage remains a challenge owing to its ‘silent’ clinical characteristics (most patients with early stage disease are asymptomatic), its difficult‐to‐access anatomical location, and its highly desmoplastic phenotype 2. Currently, surgery works better than chemotherapy, immunotherapy, and radiotherapy for HCC and CCA. However, only a small group of patients are amenable to resection or liver transplantation. To improve early diagnosis, disease progression monitoring, and postmedication evaluation of these aggressive tumors, exploiting new tests have become imperative topics.

The hepatobiliary system synthesizes the majority of plasma *N*‐glycoproteins. Studies have shown that an aberrant serum/plasma *N*‐glycome during liver cirrhosis 7, 8, 9, 10 or HCC 11, 12, 13, 14, 15, 16, 17 reflects an unhealthy status of the liver. The clinical implications of glycoscience in oncology have become clear and have impacted significantly. It is reasonable to assume that the delineation of glycosylation pattern at a single protein‐single site level may not only manifest the feature of tumors with higher sensitivity than total protein *N*‐glycome but also holds great specificity for distinguishing different hepatobiliary cancer types that are hard to pinpoint in the initial stage. Therefore, we executed a quantitative proteomic investigation with site‐specific glyco‐profiling to identify noninvasive *N*‐glycoprotein/*N*‐glycoform markers from plasma samples of patients with HCC, CCA, or combined HCC and CCA (cHCC‐CCA). From this has grown the hope that oncomedicine based on glycoproteins in liquid biopsies can be tailored in addition to conventional medical imaging.

## Materials and methods

### Study design and patients

This study was approved by the Institutional Review Board of National Cheng Kung University Hospital (NCKUH) (No. B‐ER‐103‐133). Plasma samples, clinical data, laboratory data, TNM tumor stage, and tumor differentiation grade of patients with CCA (*n* = 60), HCC (*n* = 148), and cHCC‐CCA (*n* = 12) were obtained from the Tissue Bank, Research Center of Clinical Medicine, NCKUH. All the patients were anonymized. Participants in the control group (*n* = 95), who were negative for hepatobiliary diseases, were enrolled from the Health Examination Center of NCKUH. Informed consent was obtained from each subject of the control group. All plasma samples were stored at −80°C until they are used.

### Albumin and IgG depletion, protein trypsinization, and *N*‐glycan removal

Five microliters of plasma in 100 μl of 1× phosphate‐buffered saline were incubated with 100 μl of CaptureSelect™ Human Albumin Affinity Matrix (Life Technologies, Carlsbad, CA, USA) and 50 μl of Protein G‐sepharose beads (GE Healthcare, Piscataway, NJ, USA) at room temperature for 2 h with gentle inversion. After centrifugation, the unbound proteins in supernatants were collected and kept on ice. The beads were washed with 200 μl of 1× phosphate‐buffered saline three times. All washes and the unbound proteins were combined together as the albumin‐IgG depleted fraction. Proteins that bound to the beads were eluted using 0.1 M glycine–HCl (pH 2.8) at room temperature for 10 min with vigorous vortexing as the albumin‐IgG enriched fraction. Two fractions of proteins were both denatured using 10% sodium dodecyl sulfate plus 10 mm dithiothreitol at 95°C for 10 min and alkylated with 10 mm iodoacetamide at 37°C in dark for 1 h. Salt removal and protein concentration were conducted using Amicon Ultra‐0.5 ml centrifugal filter (molecular weight cut off 3000 Da) device (Merck Millipore, Darmstadt, Germany). Devices were washed with 500 μl of deionized water three times. Concentrated proteins were quantified and half of them were treated with Peptide‐*N*‐Glycosidase F (PNGase F; New England Biolabs, Ipswich, MA, USA) at 37°C overnight to remove *N*‐glycans. Proteins with or without *N*‐glycans were then digested with sequencing grade trypsin (Promega, Fitchburg, WI, USA) in an enzyme‐to‐substrate ratio of 1:50 at 37°C overnight. The tryptic peptides were vacuum dried and stored at −80°C until they were used.

### Liquid chromatography–tandem mass spectrometry analysis

Peptides from 1.5 μg of protein samples were analyzed using Ultimate 3000 RSLC system (Dionex, Sunnyvale, CA, USA) coupled with a Q‐Exactive mass spectrometer (Thermo Fisher Scientific, Waltham, MA, USA). Mobile phase A was 0.1% fluoroacetic acid and mobile phase B was 0.1% fluoroacetic acid in 95% acetonitrile. The liquid chromatography (LC) separation was performed using a C18 column (Acclaim PepMap RSLC, 75 μm × 150 mm, 2 μm, 100 Å; Thermo Fisher Scientific) with the gradient consisting of (1) a linear increase from 1% to 25% B over 45 min, (2) a linear increase from 25% to 60% B over 10 min, and finally (3) an isocratic elution at 80% B for 10 min at 250 nl/min for separation. A full mass spectrometry (MS) scan was performed over the range of a mass‐to‐charge ratio from 300 to 2000 with a mass resolution of 140 000. The 10 most intense ions from MS scan were subjected to fragmentation for MS/MS analysis.

### Bioinformatic analysis of the glycoproteome

For protein identification, the raw LC–MS/MS data were processed into peak lists by a Proteome Discoverer 1.4 for Mascot database (version 2.4.1, Matrix Science Ltd., London, UK) search against the Swiss‐Prot_2015_07 database (548 872 sequences; 195 617 763 residues) with the following parameters: enzyme, trypsin; missed cleavages, 1; peptide mass tolerance, 10 ppm; fragment mass tolerance, 0.05 Da; fixed modification, carbamidomethyl (C); variable modification, oxidation (M), deamidated (NQ). The algorithm for protein quantification from large‐scale identification data by LC–MS/MS has been previously described 18, 19. In brief, the exponentially modified protein abundance index (emPAI), which was calculated by the number of sequenced peptides per protein, was used to relatively estimate the amount of each protein in a database search. Then, the percentage of each emPAI from the summation of the emPAI values for all of the identified proteins was used to calculate the content of each protein. For the proteins that were detected in both albumin‐IgG‐enriched and albumin‐IgG‐depleted fractions, a higher value of protein content and the associated fraction were selected. Regarding the postproteomic *N*‐glycan analysis, amino acid residues located before and after peptide sequences were merged to avoid a missing identification in the consensus motif for protein *N*‐glycosylation (Asn‐Xxx‐Ser/Thr, where Xxx can be any amino acid except proline) after the protein trypsinization. *N*‐Glycopeptides were verified by the presence of a deamidation reaction of the Asn residue on this consensus motif. GlycoPeptideSearch was used to assign glycopeptide first 20. Oxonium ions in the collision‐induced dissociation MS/MS spectra, which displayed a specific set of Y‐ions consisting of intact peptides with various attached glycan moieties, were used for the identification of glycopeptides. GlycomeDB database was then applied to confirm glycan structures attaching on the glycopeptides by searching the molecular weights of intact glycopeptides that were consistent with MS/MS spectra. All the results of site‐specific glycan analyses were checked manually. Analysts were blinded to any information about the subjects.

### Enzyme‐linked immunosorbent assay

Levels of complement C3 and galectin‐3 binding protein in plasma were measured using the Human Complement C3 ELISA kit (ab108823; Abcam, Cambridge, UK) and the Human Galectin‐3BP ELISA kit (ab213784; Abcam), respectively. The level of apolipoprotein C‐III in plasma was measured by a direct ELISA method as previously described 21.

### Statistical analysis

SPSS 18.0 for Windows (International Business Machines Corporation, Armonk, NY, USA) was used for most statistical analyses. Continuous variables were compared using Mann–Whitney *U* tests for two independent groups or Kruskal–Wallis tests with Dunn's *post hoc* tests for three or more groups. Nominal variables were compared using Fisher's exact tests or Pearson Chi‐square tests. The Pearson correlation coefficient (*r*) was used to evaluate the relationship between two factors. The analyses and Venn diagrams for proteins and peptides were obtained using InteractiVenn (http://bioinfogp.cnb.csic.es/tools/venny/) 22. Receiver operator characteristic curves were used to identify proteins expressing differentially in hepatobiliary cancers (the area under the receiver‐operating characteristic [ROC] curve >0.7 and *p* < 0.00001). Kaplan–Meier analyses and log‐rank tests were used to assess the significance of proteins on recurrence‐free survivals and overall survivals. Stepwise Cox regression analyses were used to identify factors that were associated with tumor recurrence and mortality of the patients. Significance was defined as *p* < 0.05. All *p* values were two‐tailed.

## Results

### Characteristics of the patients

There was no gender difference between each patient group and the control group (Table 1); however, a male‐predominant gender distribution was found in the HCC group when comparing to the CCA group (see supplementary material, Table S1). Three groups of patients had no age difference with each other (see supplementary material, Table S1) but they were all older than the controls (Table 1). Patients with HCC or cHCC‐CCA had abnormal alanine transaminase (ALT) and aspartate aminotransferase (AST) levels. Moreover, all groups of patients had a higher level of alkaline phosphatase (Alk‐P) and a lower level of albumin than the control group. Hematological tests revealed that all patients had a higher white blood cell count and patients with HCC or CCA had lower levels of red blood cells and hemoglobin than the controls. In addition, α‐fetoprotein level was abnormal in patients with HCC or cHCC‐CCA while carbohydrate antigen 19–9 (CA 19–9) level was abnormal in patients with CCA or cHCC‐CCA. More than 80% of the patients with HCC or cHCC‐CCA had hepatitis B or C virus infection and more than one‐fifth of them had fatty liver. Approximately 60% of the patients with HCC had been diagnosed with liver cirrhosis. Percentages of the patients with tumor stage greater than 3 were 24% in HCC, 45% in CCA, and 42% in cHCC‐CCA, respectively. More than 70% of the patients with HCC or cHCC‐CCA had recurrent tumors within a 5‐year posthepatectomy follow‐up. Five‐year survival rates in the three groups of patients were all lower than 40%.

**Table 1:** Characteristics of subjects

Variable	Control (*n* = 95)	HCC (*n* = 148)	CCA (*n* = 60)	cHCC‐CCA (*n* = 12)	*P* value 1	*P* value 2	*P* value 3
Demographic, biochemical, and hematological data							
Male, *n* (%)	61 (64.2)	110 (74.3)	31 (51.7)	9 (75.0)	0.062	0.084	0.347
Age (years)	44.0 (28.0–75.0)	60.0 (23.0–86.0)	65.5 (33.0–85.0)	59.5 (36.0–71.0)	<0.001	<0.001	<0.001
ALT (U/l)	20.0 (9.0–45.0)	51.5 (10.0–436.0)	36.5 (10.0–199.0)	44.0 (13.0–127.0)	<0.001	<0.001	<0.001
AST (U/l)	22.0 (14.0–33.0)	52.0 (17.0–800.0)	45.0 (17.0–231.0)	48.0 (28.0–205.0)	<0.001	<0.001	<0.001
Alk‐P (U/l)	59.5 (9.0–106.0)	96.0 (46.0–976.0)	138.0 (25.0–786.0)	137.5 (77.0–842.0)	<0.001	<0.001	<0.001
Albumin (g/dl)	4.7 (4.2–6.6)	4.2 (1.8–5.1)	4.2 (2.9–5.2)	4.4 (3.1–4.9)	<0.001	<0.001	<0.001
Total bilirubin (mg/dl)	0.8 (0.2–2.5)	0.6 (0.2–7.0)	0.6 (0.2–11.8)	0.6 (0.2–4.4)	<0.001	0.205	0.719
Creatinine (mg/dl)	0.9 (0.5–1.2)	0.9 (0.4–10.8)	0.8 (0.4–7.9)	0.8 (0.6–1.0)	0.481	<0.001	0.016
White blood cell (10^3^/μl)	4.8 (2.2–8.8)	5.7 (2.0–10.4)	7.0 (3.6–16.9)	6.6 (3.5–9.5)	<0.001	<0.001	0.015
Red blood cell (10^6^/μl)	4.5 (3.6–6.3)	4.2 (2.4–6.1)	4.2 (2.6–5.6)	4.4 (2.8–5.3)	<0.001	<0.001	0.210
Hemoglobin (g/dl)	14.0 (9.7–17.2)	13.4 (8.1–17.7)	12.8 (8.8–15.5)	14.4 (8.5–16.9)	<0.001	<0.001	0.980
Platelet (10^3^/μl)	212.0 (70.0–336.0)	173.0 (33.0–549.0)	215.5 (84.0–412.0)	206.5 (93.0–484.0)	<0.001	0.930	0.686
Tumor‐related factors							
α‐Fetoprotein (ng/ml)	3.7 (1.2–16.8)	38.7 (0.9–45 128.0)	3.1 (1.3–474.2)	42.8 (8.1–60 500.0)	<0.001	0.311	<0.001
CEA (ng/ml)	1.2 (0.3–4.1)	2.1 (0.4–11.1)	2.9 (0.3–60.4)	3.3 (2.2–4.4)	<0.001	<0.001	0.043
CA 19–9 (U/ml)	NA	20.0 (0.6–32 770.0)	220.0 (0.1–36 622.0)	112.7 (73.6–248.7)	NA	NA	NA
Hepatitis B, *n* (%)	0 (0.0)	84 (56.8)	19 (31.7)	8 (66.7)	<0.001	<0.001	<0.001
Hepatitis C, *n* (%)	0 (0.0)	49 (33.1)	7 (11.7)	3 (25.0)	<0.001	0.001	0.001
Fatty liver, *n* (%)	NA	34 (23.0)	NA	4 (33.3)	NA	NA	NA
Liver cirrhosis, *n* (%)	NA	90 (60.8)	NA	2 (16.7)	NA	NA	NA
Tumor staging, *n* (%)							
I	NA	58 (39.2)	11 (18.3)	2 (16.7)	NA	NA	NA
II	NA	55 (37.2)	22 (36.7)	5 (41.7)	NA	NA	NA
III	NA	31 (20.9)	6 (10.0)	3 (25.0)	NA	NA	NA
IVA	NA	2 (1.4)	18 (30.0)	2 (16.7)	NA	NA	NA
IVB	NA	2 (1.4)	3 (5.0)	0 (0.0)	NA	NA	NA
Follow‐up period (years)	NA	3.2 (0.0–11.3)	1.3 (0.1–13.2)	3.0 (0.3–7.3)	NA	NA	NA
5‐Year recurrence, *n* (%)	NA	107 (72.3)	25 (41.7)	9 (75.0)	NA	NA	NA
5‐Year survivals, *n* (%)	NA	58 (39.2)	13 (21.7)	4 (33.3)	NA	NA	NA

Data are numbers (percentages) or median values (minimum − maximum). For the patients with CCA, 10 are perihilar type and 50 are intrahepatic type. Nominal values are compared using Fisher's exact tests or Pearson Chi‐square tests. Continuous variables are compared using Mann–Whitney *U* tests. *P* value 1: comparisons between the control group and HCC group; *P* value 2: comparisons between the control group and CCA group; *P* value 3: comparisons between the control group and cHCC‐CCA group. CEA, carcinoembryonic antigen; NA, not available.

### Plasma *N*‐glycoproteome profiles in hepatobiliary cancers

**Figure 1:**
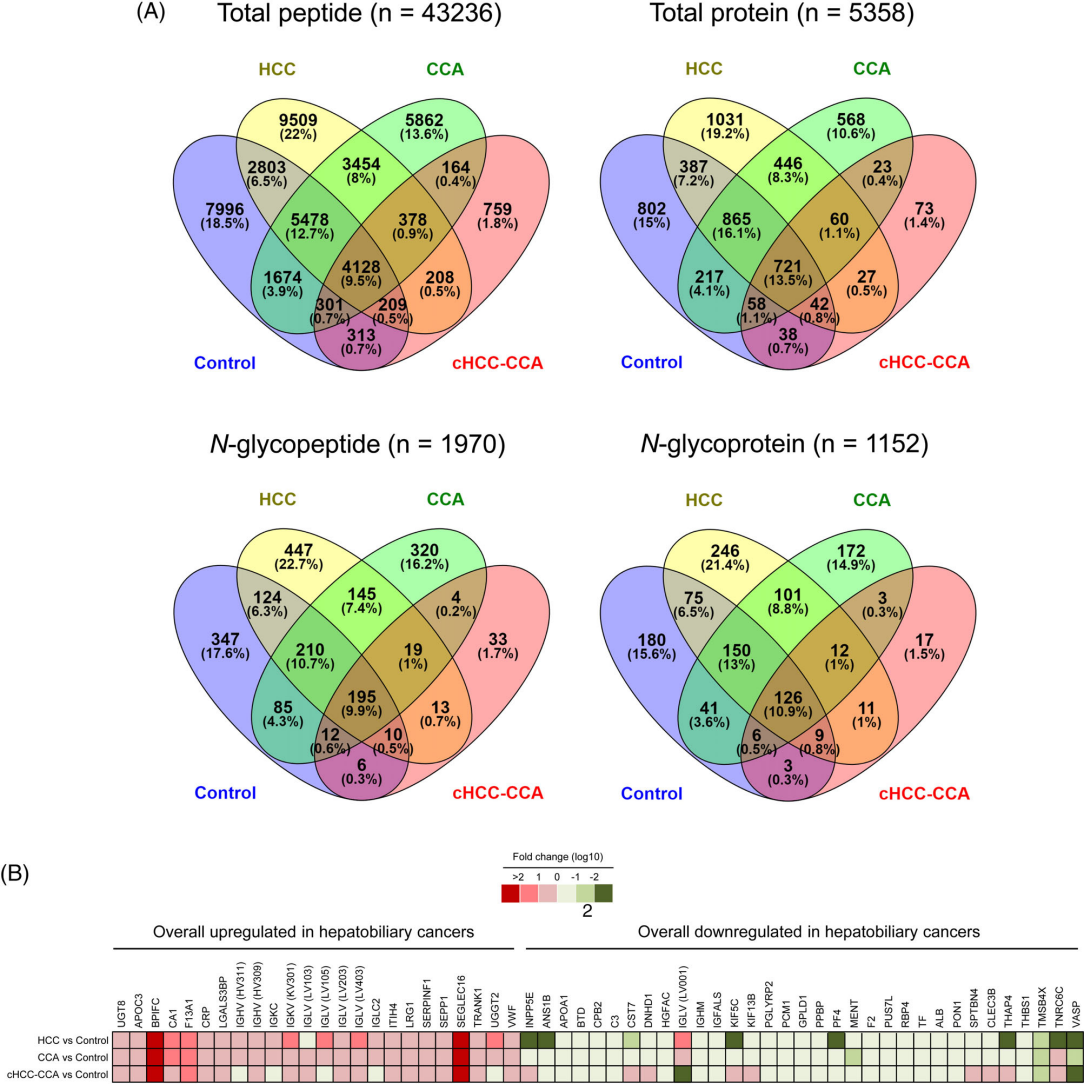
Plasma protein and peptide mapping for hepatobiliary cancers. (A) Venn diagrams for plasma proteome/peptidome and *N*‐glycoproteome/*N*‐peptidome in HCC (*n* = 148), CCA (*n* = 60), cHCC‐CCA (*n* = 12), and noncancerous controls (*n* = 95) are shown. (B) Comparisons of the content of differential proteins between the control group and different hepatobiliary cancers are shown.

A flowchart of this study is shown in supplementary material, Figure S1. A total of 43 236 peptides (Figure 1A, upper left panel) derived from 5358 proteins (Figure 1A, upper right panel), of which 721 were commonly expressed, were identified in plasma from all participants. A total of 1555 proteins were detected in both the albumin‐IgG‐enriched and albumin‐IgG‐depleted fractions. The number of common proteins was 2015 between the control and the HCC groups (Figure 1A, upper right panel; the intersection of two sets; *n* = 387 + 865 + 721 + 42), 1861 (865 + 721 + 58 + 217) between the control and the CCA groups, and 2092 (446 + 865 + 721 + 60) between the HCC and the CCA groups. However, the number of common proteins drastically decreased when the cHCC‐CCA group was included (859 [721 + 58 + 38 + 42] with the control group, 850 [721 + 42 + 27 + 60] with the HCC group, and 862 [23 + 60 + 721 + 58] with the CCA group), suggesting that the plasma proteome profile in the cHCC‐CCA group was much different from that in other groups. Among 2280 peptides with *N*‐glycosylation consensus motifs, 1970 peptides (Figure 1A, lower left panel) originated from 1152 proteins (Figure 1A, lower right panel) were *N*‐glycosylated. There were 246, 172, 17, and 180 *N*‐glycoproteins that were uniquely detected in the HCC patients, the CCA patients, the cHCC‐CCA patients, and the controls, respectively. Looking at the proteome and peptidome profiles in each group, the patients with HCC had the lowest median number of proteins (303) and peptides (2086) in plasma (see supplementary material, Figure S2). However, they had a higher percentage of *N*‐glycoproteins (49.8%) than did the controls (47.8%, *p* < 0.001) and the patients with CCA (48.0%, *p* < 0.001).

Statistical analyses (the area under the ROC curve >0.7 and *p* < 0.00001) of protein contents revealed a remarkable upregulation of 24 proteins and a downregulation of 33 proteins in hepatobiliary cancers (Table 2). The fold change of 12 differential proteins was greater than 10 when compared to the controls (Figure 1B and see supplementary material, Table S2). Overall, the transcript level of these genes in cancerous tissues corresponded with their protein contents in plasma (see supplementary material, Table S3). Thirty‐one differential proteins were detected with one or more *N*‐glycosylation sites. The contents of 12 and 9 proteins were particularly high and low in HCC, respectively (see supplementary material, Table S4). Moreover, the content of eight proteins in intrahepatic CCA differed from those in perihilar CCA (see supplementary material, Table S5). Only two of these differential proteins were slightly influenced by the age (see supplementary material, Table S6). Proteins that were affected by hepatitis B or C virus infection, liver cirrhosis, or steatosis in HCC were also illustrated (see supplementary material, Table S7).

**Table 2:** Differential proteins in hepatobiliary cancers

Gene name	Protein accession	Protein description	Identified *N*‐glycopeptide (*N*‐glycosylation site)
Upregulation in hepatobiliary cancers			
UGT8	CGT	2‐Hydroxyacylsphingosine 1‐Beta‐galactosyltransferase	RYPGIF**N**STTSDAFLQSKM **(N78)** KNLG**N**NTKL **(N333)**
APOC3	APOC3	Apolipoprotein C‐III	
BPIFC	BPIFC	BPI fold‐containing family C protein	RLALPES**N**RS **(N415)**
CA1	CAH1	Carbonic anhydrase 1	KLYPIANGN**N**QSPVDIKT **(N28)**
F13A1	F13A	Coagulation factor XIII A chain	
CRP	CRP	C‐reactive protein	
LGALS3BP	LG3BP	Galectin‐3‐binding protein	RALGFE**N**ATQALGRA **(N69)** RDAGVVCT**N**ETRS **(N125)** KEPGS**N**VTMSVDAECVPMVRD **(N192)** RFPMMLPEELFELQF**N**LSLYWSHEALFQKK **(N362)** KGL**N**LTEDTYKP **(N398)** KAAIPSALDT**N**SSKS **(N551)** RTVIRPFYLT**N**SSGVD **(N580)**
IGHV	HV311	Ig heavy chain V‐III region KOL	
	HV309	Ig heavy chain V‐III region NIE	
IGKC	IGKC	Ig kappa chain C region	
IGKV	KV301	Ig kappa chain V‐III region B6	
IGLV	LV103	Ig lambda chain V‐I region NEW	
	LV105	Ig lambda chain V‐I region NEWM	
	LV203	Ig lambda chain V‐II region BOH	
	LV403	Ig lambda chain V‐IV region Hil	
IGLC2	LAC2	Ig lambda‐2 chain C regions	
ITIH4	ITIH4	Inter‐alpha‐trypsin inhibitor heavy chain H4	KAFIT**N**FSMIIDGMTYPGIIKE **(N81)** KHLQMDIHIFEPQGISFLETESTFMTNQLVDALTTWQ **N**KT **(N207)** KLPTQ**N**ITFQTESSVAEQEAEFQSPKY **(N517)** RNQAL**N**LSLAYSFVTPLTSMVVTKP **(N577)**
LRG1	A2GL	Leucine‐rich alpha‐2‐glycoprotein	RSDHGSSISCQPPAEIPGYLPADTVHLAVEFF**N**LTHLPANLLQGASKL **(N79)** KLPPGLLA**N**FTLLRT **(N186)** RQLDMLDLS**N**NSLASVPEGLWASLGQPNWDMRD **(N269)** RDGFDISGNPWICDQ**N**LSDLYRW **(N306)** KMFSQ**N**DTRC **(N325)**
SERPINF1	PEDF	Pigment epithelium‐derived factor	KVTQ**N**LTLIEESLTSEFIHDIDRE **(N285)**
SEPP1	SEPP1	Selenoprotein P	RDQDPMLNS**N**GSVTVVALLQASUYLCILQASKL **(N46)** KVSEHIPVYQQEE**N**QTDVWTLL**N**GSKD **(N119, N128)**
SIGLEC16	SIG16	Sialic acid‐binding Ig‐like lectin 16	
TRANK1	TRNK1	TPR and ankyrin repeat‐containing protein 1	RDLAVLLC**N**KS **(N20)** K**N**DSLLLAWNKA **(N594)**
UGGT2	UGGG2	UDP‐glucose: glycoprotein glucosyltransferase 2	KGIVENMGINAN**N**MSDFIMKV **(N920)**
VWF	VWF	von Willebrand factor	RASPPSSSC**N**ISSGEMQKG **(N211)** KIGEADF**N**RS **(N1515)** KV**N**CTTQPCPTAKA **(N2290)** RGLQPTLTNPGECRP**N**FTCACRK **(N2357)** RMEACML**N**GTVIGPGKT **(N2585)** KEE**N**NTGECCGRC **(N2635)**
Downregulation in hepatobiliary cancers			
INPP5E	INP5E	72 kDa inositol polyphosphate 5‐phosphatase	
ANKS1B	ANS1B	Ankyrin repeat and sterile alpha motif domain‐containing protein 1B	KSNQLE**N**HTIVGTRS **(N683)**
APOA1	APOA1	Apolipoprotein A‐I	
BTD	BTD	Biotinidase	RF**N**DTEVLQRL **(N150)** KNPVGLIGAE**N**ATGETDPSHSKF **(N349)**
CPB2	CBPB2	Carboxypeptidase B2	KQVHFFV**N**ASDVDNVKA **(N73)** KAHLNVSGIPCSVLLADVEDLIQQQIS**N**DTVSPRA **(N108)**
C3	CO3	Complement C3	KTVLTPATNHMG**N**VTFTIPANRE **(N85)** RM**N**KT **(N939)**
CST7	CYTF	Cystatin‐F	
DNHD1	DNHD1	Dynein heavy chain domain‐containing protein 1	KSSFL**N**RS **(N2264)**
HGFAC	HGFA	Hepatocyte growth factor activator	RCFLG**N**GTGYRG **(N290)**
IGLV	LV001	Ig lambda chain V region 4A	
IGHM	IGHM	Ig mu chain C region	RGLTFQQ**N**ASSMCGPDQDTAIRV **(N209)** KTHT**N**ISESHP**N**ATFSAVGEASICEDDWDSGERF **(N272 or N279)** KPTLY**N**VSLVMSDTAGTCY **(N440)**
IGFALS	ALS	Insulin‐like growth factor‐binding protein complex acid labile subunit	R**N**NSLRT **(N515)** RFVQAICEGDDCQPPAYTYN**N**ITCASPPEVVGLDLRD **(N580)**
KIF5C	KIF5C	Kinesin heavy chain isoform 5C	KSLV**N**RS **(N603)**
KIF13B	KI13B	Kinesin‐like protein KIF13B	REATL**N**NSLMRL **(N680)**
PGLYRP2	PGRP2	*N*‐Acetylmuramoyl‐l‐alanine amidase	RLEPVHLQLQCMSQEQLAQVAA**N**ATKE **(N367)**
PCM1	PCM1	Pericentriolar material 1 protein	RI**N**FSDLDQRS **(N108)** RKPFNFLPMQINT**N**KS **(N151)** RTVNSNCEIN**N**RS **(N586)** KQNS**N**NTRG **(N711)** RGNA**N**KT **(N718)** RQQ**N**ISMQRQ **(N975)**
GPLD1	PHLD	Phosphatidylinositol‐glycan‐specific phospholipase D	RNI**N**YTERG **(N321)** KLNVEAA**N**WTVRG **(N568)** KLGTSLSSGHVLM**N**GTLKQ **(N659)**
PPBP	CXCL7	Platelet basic protein	
PF4	PLF4	Platelet factor 4	
MENT	MENT	Protein MENT	
F2	THRB	Prothrombin	RGHV**N**ITRS **(N121)** KPEI**N**STTHPGADLQENFCRN **(N143)** RSEGSSV**N**LSPPLEQCVPDRG **(N205)**
PUS7L	PUS7L	Pseudouridylate synthase 7 homolog‐like protein	KQI**N**DSANLRE **(N404)**
RBP4	RET4	Retinol‐binding protein 4	
TF	TRFE	Serotransferrin	KCGLVPVLAENY**N**KS **(N432)** RQQQHLFGS**N**VTDCSGNFCLFRS **(N630)**
ALB	ALBU	Serum albumin	
PON1	PON1	Serum paraoxonase/arylesterase 1	RVVAEGFDFANGI**N**ISPDGKY **(N227)** KHA**N**WTLTPLKS **(N253)** KSLDFNTLVD**N**ISVDPETGDLWVGCHPNGMKI **(N279)** KVTQVYAE**N**GTVLQGSTVASVYKG **(N324)**
SPTBN4	SPTN4	Spectrin beta chain, nonerythrocytic 4	
CLEC3B	TETN	Tetranectin	
THAP4	THAP4	THAP domain‐containing protein 4	RD**N**WTPTKY **(N46)** RQ**N**KS **(N355)**
THBS1	TSP1	Thrombospondin‐1	KGCSSSTSVLLTLDNNVV**N**GSSPAIRT **(N248)** KVSCPIMPCS**N**ATVPDGECCPRC **(N360)** KVV**N**STTGPGEHLRN **(N1067)**
TMSB4X	TYB4	Thymosin beta‐4	
TNRC6C	TNR6C	Trinucleotide repeat‐containing gene 6C protein	MATGSAQG**N**FTGHTKK **(N9)** KQ**N**GSSSAVQKE **(N282**) R**N**VSGSMRQ **(N1176)** R**N**LTPQIDGSTLRT **(N1520)**
VASP	VASP	Vasodilator‐stimulated phosphoprotein	

Protein content [molecular %; exponentially emPAI/Σ(emPAI) × 100] was used for the protein semi‐quantification. ROC analysis (the area under the ROC curve >0.7 and *p* < 0.00001) is used to identify proteins that are differentially expressed in hepatobiliary carcinoma or CCA. *N*‐glycosylation sites on peptides are shown in bold font.

### Essential factors for tumor stage, differentiation, and prognosis

**Figure 2:**
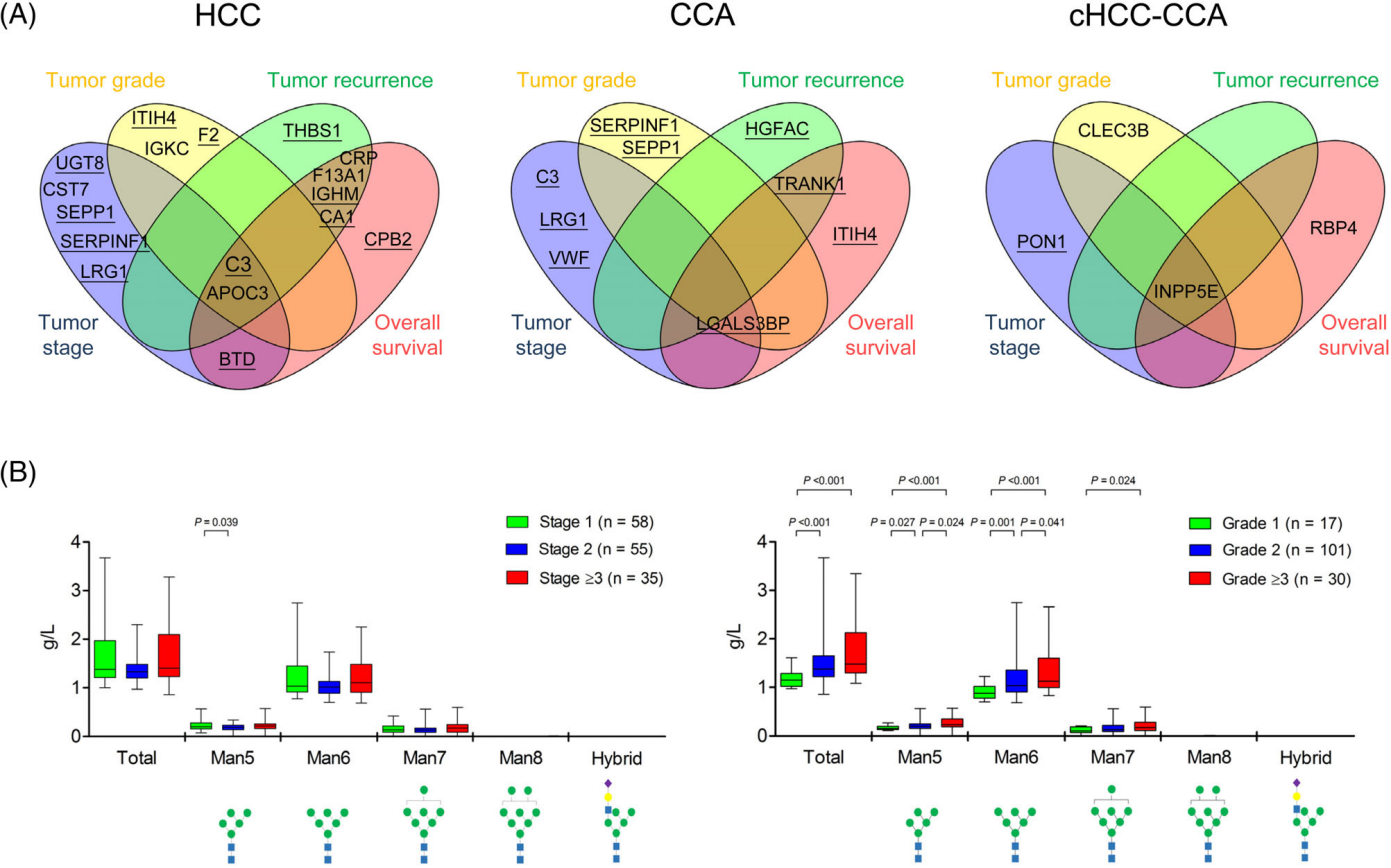
Plasma *N*‐glycoprotein/*N*‐glycoform markers of hepatobiliary cancers. (A) Plasma protein markers for HCC, CCA, and cHCC‐CCA are shown in Venn diagrams. *N*‐glycoproteins are underlined. Please refer to Table 2 for the full name of each protein. (B) An association of the level of plasma complement C3 bearing different glycoforms with HCC tumor grade is shown as box‐and‐whisker plots. *P* values are obtained from Kruskal–Wallis tests with Dunn's *post hoc* tests.

We next assessed the clinical relevance of the protein contents by emPAI % value of these 57 candidates in different hepatobiliary cancers. Proteins that were associated with the tumor stage, tumor grade, recurrence‐free survival, and overall survival of HCC, CCA, and cHCC‐CCA, respectively, are shown in Figure 2A and see supplementary material, Figures S3 and S4. Of note, the emPAI % values of complement C3 and apolipoprotein C‐III were associated with the tumor progression and prognosis of HCC, as galectin‐3‐binding protein in CCA and 72 kDa inositol polyphosphate 5‐phosphatase in cHCC‐CCA. Strong correlations between emPAI % values and actual amounts of these essential proteins were observed (see supplementary material, Figure S5).

### Site‐specific *N*‐glycan profiling

**Figure 3:**
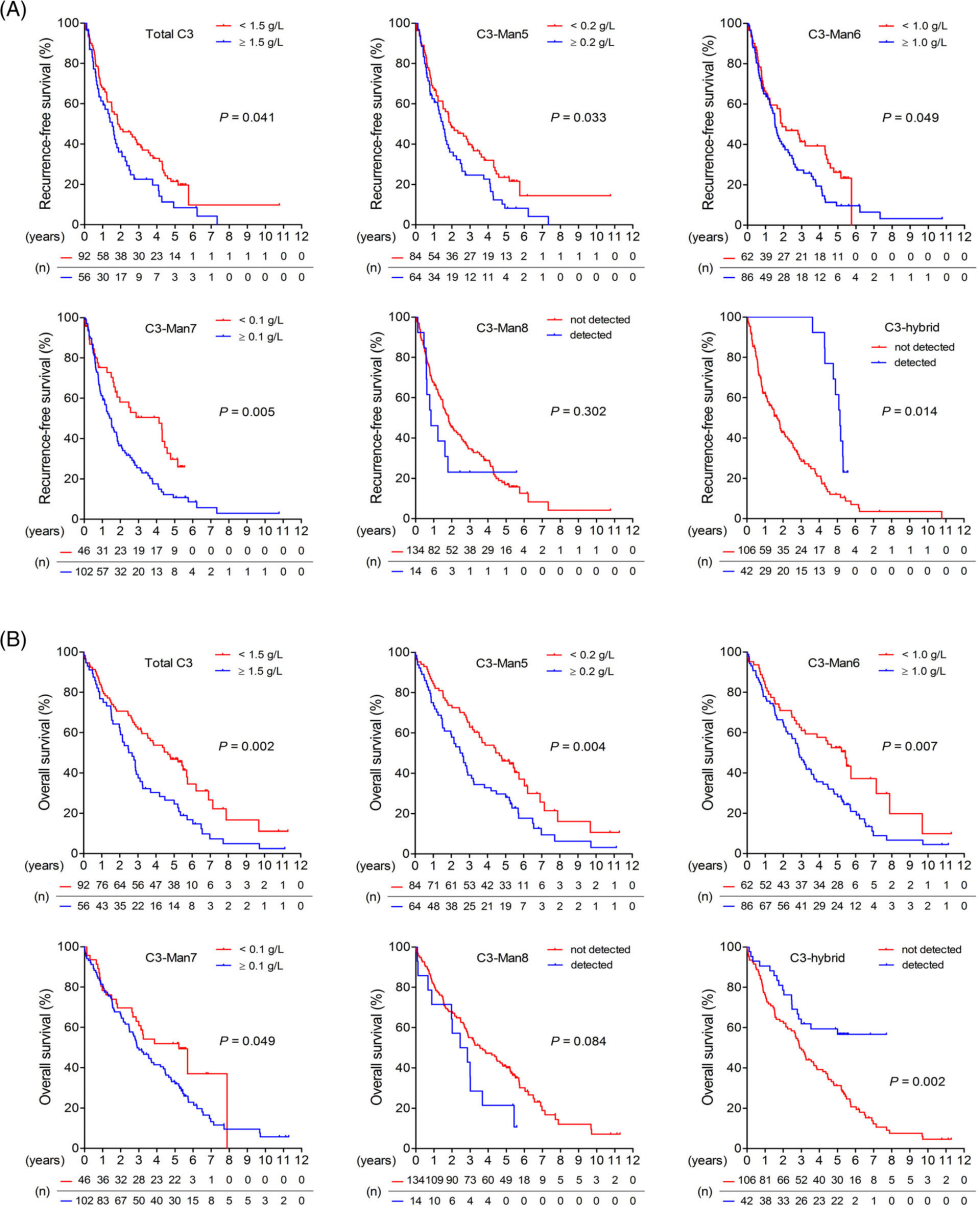
The relevance of different complement C3 glycoforms in the prognosis of HCC**.** Kaplan–Meier analyses of associations between complement C3 bearing different glycoforms with (A) recurrence‐free survivals and (B) overall survivals in patients with HCC (*n* = 148) are shown. *P* values are obtained from log‐rank tests.

Apolipoprotein C‐III and 72 kDa inositol polyphosphate 5‐phosphatase were not *N*‐glycosylated. An *N*‐glycan structure analysis was performed only for the glycopeptides containing Asn85 of complement C3 and was excluded for glycopeptides containing Asn939 of complement C3 and glycopeptides corresponding to galectin‐3‐binding protein because of weak signal intensities of these fragments in the mass spectra. In regard to the glycosylation pattern of complement C3 Asn85, the patients with HCC or cHCC‐CCA had a higher proportion of Hex7HexNAc2 (mannose‐7; Man7) glycoform than the patients with CCA and the controls (see supplementary material, Table S8). Furthermore, when compared with the controls, all the patient groups had lower proportions of Hex5HexNAc2 (mannose‐5; Man5) and Hex6HexNAc3SA1 (hybrid) glycoforms. We next analyzed the clinical relevance of each glycoform of at Asn85 of complement C3 in HCC. The proportion of each glycovariant in the patients with HCC was shown in supplementary material, Table S9. The concentration of complement C3 with Man5, Man6, or Man7 glycoform at Asn85 closely linked to the tumor grade (Figure 2B) and the association was stronger than did α‐fetoprotein, a renowned HCC biomarker (see supplementary material, Figure S6). The glycoprofile of complement C3 Asn85 was independent to the age (see supplementary material, Table S10). Results from Kaplan–Meier analyses showed that levels of total complement C3 protein and certain C3 glycovariants were associated with the recurrence rate and the mortality rate of HCC (Figure 3A,B). Stepwise Cox regression analyses revealed that tumor stage, AST, complement C3 with Man5 glycoform, and complement C3 with hybrid glycoform were independent factors for the recurrent HCC (Table 3). Furthermore, tumor stage, albumin, liver cirrhosis, and complement C3 with hybrid glycoform were associated with the mortality rate of HCC. The correlation of complement C3 bearing Man5 or hybrid glycoform with the postsurgery prognosis of HCC was stronger than the total complement C3 protein level (Table 3).

**Table 3:** Cox regression analyses of recurrence and mortality rates of HCC

Variable	Recurrence rate				Mortality rate			
	Univariate		Multivariate		Univariate		Multivariate	
	Hazard ratio (95% CI)	*P* value	Hazard ratio (95% CI)	*P* value	Hazard ratio (95% CI)	*P* value	Hazard ratio (95% CI)	*P* value
Sex (male = 1, female = 0)	0.997 (0.979–1.015)	0.749			1.003 (0.984–1.021)	0.775		
Age (years)	0.879 (0.571–1.352)	0.556			0.975 (0.631–1.507)	0.909		
Tumor stage	1.393 (1.078–1.801)	0.011	1.447 (1.039–2.014)	0.029	1.519 (1.177–1.960)	0.001	1.457 (1.010–2.102)	0.044
Tumor grade	1.453 (1.044–2.022)	0.027	1.214 (0.774–1.903)	0.398	1.470 (1.055–2.049)	0.023	1.392 (0.808–2.398)	0.234
Albumin (g/dl)	0.798 (0.612–1.041)	0.096			0.670 (0.527–0.851)	0.001	0.548 (0.361–0.830)	0.005
Alanine aminotransferase (U/l)	1.001 (0.999–1.004)	0.403			1.001 (0.999–1.003)	0.377		
Aspartate aminotransferase (U/l)	1.005 (1.002–1.008)	0.002	1.004 (1.001–1.008)	0.022	1.004 (1.002–1.007)	<0.001	1.002 (0.999–1.006)	0.118
Red blood cell (10^6^/μl)	0.769 (0.546–1.083)	0.133			0.722 (0.514–1.014)	0.060		
White blood cell (10^3^/μl)	0.988 (0.879–1.110)	0.893			1.050 (0.940–1.173)	0.387		
Platelet (10^3^/μl)	0.998 (0.996–1.001)	0.127			1.000 (0.997–1.002)	0.752		
Hepatitis B virus infection (yes = 1, no = 0)	1.089 (0.744–1.593)	0.662			0.750 (0.515–1.093)	0.135		
Hepatitis C virus infection (yes = 1, no = 0)	1.287 (0.864–1.918)	0.214			1.175 (0.797–1.732)	0.414		
Liver cirrhosis (yes = 1, no = 0)	1.557 (1.042–2.326)	0.031	1.656 (0.992–2.765)	0.054	1.517 (1.016–2.266)	0.041	1.970 (1.058–3.667)	0.033
Fatty liver (yes = 1, no = 0)	0.688 (0.426–1.110)	0.125			0.666 (0.404–1.097)	0.110		
Total complement C3 ≥ 1.5 g/l (yes = 1, no = 0)	1.490 (1.014–2.189)	0.042	1.050 (0.562–1.961)	0.878	1.786 (1.225–2.603)	0.003	1.189 (0.571–2.473)	0.644
Complement C3‐Man5 ≥ 0.2 g/l (yes = 1, no = 0)	1.499 (1.030–2.183)	0.035	1.768 (1.011–3.092)	0.046	1.718 (1.181–2.500)	0.005	1.503 (0.783–2.884)	0.220
Complement C3‐Man6 ≥ 1.0 g/l (yes = 1, no = 0)	1.473 (0.998–2.174)	0.051			1.718 (1.151–2.563)	0.008	0.978 (0.469–2.043)	0.954
Complement C3‐Man7 ≥ 0.1 g/l (yes = 1, no = 0)	1.851 (1.202–2.852)	0.005	1.343 (0.780–2.315)	0.288	1.567 (0.999–2.459)	0.051		
Complement C3‐Man8 detected (yes = 1, no = 0)	1.409 (0.733–2.708)	0.304			1.698 (0.926–3.114)	0.087		
Complement C3‐hybrid detected (yes = 1, no = 0)	0.579 (0.371–0.902)	0.016	0.551 (0.317–0.957)	0.034	0.451 (0.271–0.751)	0.002	0.421 (0.200–0.886)	0.023

## Discussion

Hepatobiliary cancer is highly progressive. Despite a wide array of tumor markers and treatment options, the prognosis of hepatobiliary cancer remains poor. Recent advances in glycan‐detection approaches have accelerated interest in clinical glycoproteomics for the discovery of detection markers or therapeutic targets for chronic disease and cancer. Here, we identified circulating *N*‐glycoprotein/*N*‐glycoform markers to help early diagnosis, monitoring of disease progression, and evaluation of medication outcomes of HCC, CCA, and cHCC‐CCA.

One major challenge in mass spectrometry‐based clinical glycoproteomics is to quantify native proteins in specimens in a label‐free manner. Several algorithms have been proposed to estimate protein‐abundance accompanied by large‐scale validation results including the spectral count and its derivatives 23, 24, 25. Rappsilber *et al* first posed the PAI method 26, which evaluates the number of peptides observed from a protein relative to the total number of observable peptides. However, the length and amino acid composition of peptides, and ionization efficiency, and so on may disturb the observability of peptide fragments by the mass spectrometer. Later reported by Ishihama *et al*, emPAI estimated protein amount in proteomics by the number of sequenced peptides per protein 18 and showed a satisfactory correlation with the actual protein amount in complex mixtures. In 2010, Shinoda *et al* presented emPAI %, a powerful and accurate calculation method for acquiring a relative content of individual proteins 19. Herein, complement C3, apolipoprotein C‐III, and galectin‐3‐binding protein were selected under this algorithm and their emPAI % values showed a high correspondence to the actual protein concentrations. Using emPAI % as a screening platform, though it may result in a loss of target detection, holds great potentials for the application of plasma proteome to routine laboratory tests, especially when we currently have not been able to quantify whole proteins in specimens.

We identified 57 differential proteins in hepatobiliary cancers. It is easy to understand the downregulation of proteins produced by the liver, such as albumin and serotransferrin, as a result of impaired liver function during hepatocarcinogenesis. We also observed the upregulation of two glycosylation‐related enzymes, UGT8, and UGGT2, in the plasma samples of the patients. UGT8 promotes the biosynthesis of galactocerebrosides, which are abundant sphingolipids of the myelin membrane of the central and peripheral nervous systems. UGGT2 transfers a glucose monomer to the misfolded glycoproteins, thus providing quality control for protein transport out of the endoplasmic reticulum. Currently, there is no evidence to prove a relationship between hepatobiliary cancers and UGT8 or UGGT2. However, indirect effects of UGT8 and UGGT2 upregulation on the change of the liver microenvironment and tumorigenic events of hepatocytes or cholangiocytes might be suspected.

Four proteins closely related to tumor progression and prognosis of hepatobiliary malignancies were found. Galectin‐3‐binding protein, also named MAC‐2‐binding protein, is known to mediate cell‐to‐cell adhesion and initiate pathologic, proinflammatory responses 27, 28, 29. Enhanced galectin‐3‐binding protein expression has been linked to poor prognosis in colorectal cancer, diffuse large B‐cell lymphoma, and lung cancer 30, 31, 32, 33. It has also been reported to be an accurate diagnostic marker for CCA 34, which is consistent with our finding. A novel marker we identified for cHCC‐CCA is 72 kDa inositol polyphosphate 5‐phosphatase, which is involved in intracellular calcium mobilization, insulin‐related signal transduction, and glucose homeostasis. The pathological roles and detailed mechanisms of 72 kDa inositol polyphosphate 5‐phosphatase on cHCC‐CCA need to be further addressed. Regarding HCC, the first marker apolipoprotein C‐III is a major structural component of very‐low‐density lipoprotein but is also present in chylomicrons and high‐density lipoprotein. It inhibits lipoprotein lipase and hepatic lipase and promotes the assembly and secretion of very‐low‐density lipoprotein particles from hepatic cells 35. Apolipoprotein C‐III has attracted much attention owing to its relationship with hyperlipoproteinemia and fatty liver disease but yet directly touches HCC. The other HCC marker complement C3 is a front and center factor of classical, alternative, or lectin pathways of the complement system. Cleaved complement C3 triggers activation of the complement cascade, which augments host immune functions including lysis of bacteria and cells by forming membrane‐attack complex, opsonization, and chemotaxis of leukocytes 36. It is not surprising that downregulation of complement C3 was detected in patients with HCC 37, 38 because of their compromised immune system during hepatocarcinogenesis. However, beyond empirical speculation, we found that the level of complement C3 correlated positively with poor differentiation of tumor cells and an unfavorable prognosis of HCC. A growing body of evidence supports roles for activated components of the complement system in various aspects of carcinogenesis, including chronic inflammation, tumor immunoescape, tumor cell proliferation, angiogenesis, and tumor invasion 39, 40, 41. Moreover, complement inhibition‐related therapeutic strategies for cancer treatment have been designed 42, 43. Given the above, it could be expected that complement C3‐targeted inhibitors, such as APL‐2 and compstatin, may find application in cancer pharmaceutics in the future.

The postproteomic glycan analysis herein primarily focused on Asn85 of complement C3 because apolipoprotein C‐III and 72 kDa inositol polyphosphate 5‐phosphatase were not *N*‐glycoproteins. Moreover, other glycopeptide fragments belonging to complement C3 and galectin‐3 binding protein did not possess enough signal intensity for high‐resolution glycan analyses under a whole plasma proteome. Our data are akin to previous reports showing that mainly high‐mannose sugar chains cover human complement C3 protein; Man5 or Man6 on Asn85 and Man8 or Man9 on Asn 44, 45, 46. One can easily understand that the *C3* gene is highly conserved among species owing to its importance in the immune system 47. Nonetheless, it is intriguing to detect human complement C3 proteins that are equipped with high‐mannose glycans since high‐mannose type structures on mature proteins are usually present in lower eukaryotes but are rarely found in higher eukaryotes except the precursor oligosaccharides during glycan biosynthesis. More studies are needed to comprehend why high‐mannose *N*‐glycans are retained in the human complement C3 protein. In addition, a secondary structural model proposes that all three Asn residues on complement C3 are part of reverse turns 48. Accordingly, it is plausible to assume that alteration of glycan composition at Asn85 in patients with HCC has a profound effect on the interaction of complement C3 with other factors, thereby contributing to hepatocellular carcinogenesis.

Taken together, our findings, in the context of HCC, CCA, and cHCC‐HCC, may enable new insight and foresight on the diagnosis, monitoring of tumor progression, and prognosis of different hepatobiliary cancers. With the continued emergence of new biotechnologies beyond the realm of the glycoproteome, validation cohorts, even clinical application, of inhibitors or antagonists of these tumor markers for the treatment of hepatobiliary cancers may be developed soon.

## Author contributions statement

T‐TC was responsible for experimental design and coordinated the study. J‐HC and S‐YH carried out bioinformatic analyses. H‐WT was responsible for histological data. K‐CY assisted in ELISA‐based protein quantification. C‐HH was responsible for the experimental performance, data analyses, and manuscript writing.

## Availability of data and material

The mass spectrometry proteomics data have been deposited to the ProteomeXchange Consortium *via* the PRIDE partner repository with the dataset identifier (Accession number: PXD013629; Username: reviewer19947@ebi.ac.uk; Password: 7Lmoj8AB). Other data are available from the corresponding author on reasonable request.

## Supporting information


**Figure S1.** Flowchart of the study design
**Figure S2.** Comparisons of plasma proteome/peptidome and *N*‐glycoproteome/*N*‐glycopeptidome among HCC, CCA, cHCC‐CCA, and controls.
**Figure S3.** Factors associated with tumor stage and grade
**Figure S4.** Kaplan–Meier analyses of recurrence‐free and overall survival
**Figure S5.** Correlations between protein content and protein concentration of complement C3, apolipoprotein C‐III, and galectin‐3‐binding protein
**Figure S6.** Levels of α‐fetoprotein in different tumor grades

**Table S1.** Comparisons of characteristics of the patients with different types of hepatobiliary cancer

**Table S2.** Comparison of differential protein content between patients with hepatobiliary cancers and controls

**Table S3.** Changes in mRNA levels of differential proteins in hepatobiliary cancers

**Table S4.** Comparison of differential protein content among different hepatobiliary cancers

**Table S5.** Comparison of differential protein content between patients with intrahepatic CCA and patients with perihilar CCA

**Table S6.** Relationship between age and differential protein content in patients with hepatobiliary cancers

**Table S7.** Differential protein expression in patients with hepatocellular carcinoma with different risk factors

**Table S8.** Complement C3 asparagine85 glycoprofiles

**Table S9.** Complement C3 Asparagine85 glycoprofiles in patients with hepatocellular carcinoma

**Table S10.** Relationship between age and complement C3 asparagine85 glycoprofile in patients with hepatocellular carcinoma

## References

[1] Byam J , Renz J , Millis JM . Liver transplantation for hepatocellular carcinoma. Hepatobiliary Surg Nutr 2013; 2: 22–30. 24570911 10.3978/j.issn.2304-3881.2012.11.03 PMC3924634

[2] Rizvi S , Khan SA , Hallemeier CL , *et al* Cholangiocarcinoma – evolving concepts and therapeutic strategies. Nat Rev Clin Oncol 2018; 15: 95–111. 28994423 10.1038/nrclinonc.2017.157 PMC5819599

[3] Shin HR , Oh JK , Masuyer E , *et al* Comparison of incidence of intrahepatic and extrahepatic cholangiocarcinoma – focus on east and south‐eastern Asia. Asian Pac J Cancer Prev 2010; 11: 1159–1166. 21198257

[4] Ustundag Y , Bayraktar Y . Cholangiocarcinoma: a compact review of the literature. World J Gastroenterol 2008; 14: 6458–6466. 19030196 10.3748/wjg.14.6458 PMC2773330

[5] Poomphakwaen K , Promthet S , Kamsa‐Ard S , *et al* Risk factors for cholangiocarcinoma in Khon Kaen, Thailand: a nested case‐control study. Asian Pac J Cancer Prev 2009; 10: 251–258. 19537893

[6] Blechacz B , Komuta M , Roskams T , *et al* Clinical diagnosis and staging of cholangiocarcinoma. Nat Rev Gastroenterol Hepatol 2011; 8: 512–522. 21808282 10.1038/nrgastro.2011.131 PMC3331791

[7] Callewaert N , Van Vlierberghe H , Van Hecke A , *et al* Noninvasive diagnosis of liver cirrhosis using DNA sequencer‐based total serum protein glycomics. Nat Med 2004; 10: 429–434. 15152612 10.1038/nm1006

[8] Klein A , Michalski JC , Morelle W . Modifications of human total serum N‐glycome during liver fibrosis–cirrhosis, is it all about immunoglobulins? Proteomics Clin Appl 2010; 4: 372–378. 21137057 10.1002/prca.200900151

[9] Morelle W , Flahaut C , Michalski JC , *et al* Mass spectrometric approach for screening modifications of total serum *N*‐glycome in human diseases: application to cirrhosis. Glycobiology 2006; 16: 281–293. 16339757 10.1093/glycob/cwj067

[10] Ocho M , Togayachi A , Iio E , *et al* Application of a glycoproteomics‐based biomarker development method: alteration in glycan structure on colony stimulating factor 1 receptor as a possible glycobiomarker candidate for evaluation of liver cirrhosis. J Proteome Res 2014; 13: 1428–1437. 24422531 10.1021/pr400986t

[11] Huang Y , Wu H , Xue R , *et al* Identification of N‐glycosylation in hepatocellular carcinoma patients' serum with a comparative proteomic approach. PLoS One 2013; 8: e77161. 24143209 10.1371/journal.pone.0077161 PMC3797089

[12] Yang G , Cui T , Wang Y , *et al* Selective isolation and analysis of glycoprotein fractions and their glycomes from hepatocellular carcinoma sera. Proteomics 2013; 13: 1481–1498. 23436760 10.1002/pmic.201200259

[13] Kaji H , Ocho M , Togayachi A , *et al* Glycoproteomic discovery of serological biomarker candidates for HCV/HBV infection‐associated liver fibrosis and hepatocellular carcinoma. J Proteome Res 2013; 12: 2630–2640. 23586699 10.1021/pr301217b

[14] Block TM , Comunale MA , Lowman M , *et al* Use of targeted glycoproteomics to identify serum glycoproteins that correlate with liver cancer in woodchucks and humans. Proc Natl Acad Sci U S A 2005; 102: 779–784. 15642945 10.1073/pnas.0408928102 PMC545516

[15] Comunale MA , Lowman M , Long RE , *et al* Proteomic analysis of serum associated fucosylated glycoproteins in the development of primary hepatocellular carcinoma. J Proteome Res 2006; 5: 308–315. 16457596 10.1021/pr050328x

[16] Ang IL , Poon TC , Lai PB , *et al* Study of serum haptoglobin and its glycoforms in the diagnosis of hepatocellular carcinoma: a glycoproteomic approach. J Proteome Res 2006; 5: 2691–2700. 17022640 10.1021/pr060109r

[17] Gao HJ , Chen YJ , Zuo D , *et al* Quantitative proteomic analysis for high‐throughput screening of differential glycoproteins in hepatocellular carcinoma serum. Cancer Biol Med 2015; 12: 246–254. 26487969 10.7497/j.issn.2095-3941.2015.0010 PMC4607824

[18] Ishihama Y , Oda Y , Tabata T , *et al* Exponentially modified protein abundance index (emPAI) for estimation of absolute protein amount in proteomics by the number of sequenced peptides per protein. Mol Cell Proteomics 2005; 4: 1265–1272. 15958392 10.1074/mcp.M500061-MCP200

[19] Shinoda K , Tomita M , Ishihama Y . emPAI Calc – for the estimation of protein abundance from large‐scale identification data by liquid chromatography‐tandem mass spectrometry. Bioinformatics 2010; 26: 576–577. 20031975 10.1093/bioinformatics/btp700

[20] Chandler KB , Pompach P , Goldman R , *et al* Exploring site‐specific *N*‐glycosylation microheterogeneity of haptoglobin using glycopeptide CID tandem mass spectra and glycan database search. J Proteome Res 2013; 12: 3652–3666. 23829323 10.1021/pr400196s PMC3777712

[21] Sun HY , Lin CC , Lee JC , *et al* Very low‐density lipoprotein/lipo‐viro particles reverse lipoprotein lipase‐mediated inhibition of hepatitis C virus infection via apolipoprotein C‐III. Gut 2013; 62: 1193–1203. 22689516 10.1136/gutjnl-2011-301798

[22] Heberle H , Meirelles GV , da Silva FR , *et al* InteractiVenn: a web‐based tool for the analysis of sets through Venn diagrams. BMC Bioinformatics 2015; 16: 169. 25994840 10.1186/s12859-015-0611-3 PMC4455604

[23] Liu H , Sadygov RG , Yates JR III . A model for random sampling and estimation of relative protein abundance in shotgun proteomics. Anal Chem 2004; 76: 4193–4201. 15253663 10.1021/ac0498563

[24] Lu P , Vogel C , Wang R , *et al* Absolute protein expression profiling estimates the relative contributions of transcriptional and translational regulation. Nat Biotechnol 2007; 25: 117–124. 17187058 10.1038/nbt1270

[25] Zybailov B , Mosley AL , Sardiu ME , *et al* Statistical analysis of membrane proteome expression changes in Saccharomyces cerevisiae . J Proteome Res 2006; 5: 2339–2347. 16944946 10.1021/pr060161n

[26] Rappsilber J , Ryder U , Lamond AI , *et al* Large‐scale proteomic analysis of the human spliceosome. Genome Res 2002; 12: 1231–1245. 12176931 10.1101/gr.473902 PMC186633

[27] Filer A , Bik M , Parsonage GN , *et al* Galectin 3 induces a distinctive pattern of cytokine and chemokine production in rheumatoid synovial fibroblasts via selective signaling pathways. Arthritis Rheum 2009; 60: 1604–1614. 19479862 10.1002/art.24574 PMC3116228

[28] Newlaczyl AU , Yu LG . Galectin‐3 – a jack‐of‐all‐trades in cancer. Cancer Lett 2011; 313: 123–128. 21974805 10.1016/j.canlet.2011.09.003

[29] Silverman AM , Nakata R , Shimada H , *et al* A galectin‐3‐dependent pathway upregulates interleukin‐6 in the microenvironment of human neuroblastoma. Cancer Res 2012; 72: 2228–2238. 22389450 10.1158/0008-5472.CAN-11-2165 PMC3815584

[30] Wu CC , Huang YS , Lee LY , *et al* Overexpression and elevated plasma level of tumor‐associated antigen 90K/mac‐2 binding protein in colorectal carcinoma. Proteomics Clin Appl 2008; 2: 1586–1595. 21136809 10.1002/prca.200800080

[31] Kim SJ , Lee SJ , Sung HJ , *et al* Increased serum 90K and galectin‐3 expression are associated with advanced stage and a worse prognosis in diffuse large B‐cell lymphomas. Acta Haematol 2008; 120: 211–216. 19153476 10.1159/000193223

[32] Ozaki Y , Kontani K , Teramoto K , *et al* Involvement of 90K/Mac‐2 binding protein in cancer metastases by increased cellular adhesiveness in lung cancer. Oncol Rep 2004; 12: 1071–1077. 15492795

[33] Iacovazzi PA , Notarnicola M , Caruso MG , *et al* Serum levels of galectin‐3 and its ligand 90k/Mac‐2BP in colorectal cancer patients. Immunopharmacol Immunotoxicol 2010; 32: 160–164. 19686089 10.1080/08923970902936880

[34] Koopmann J , Thuluvath PJ , Zahurak ML , *et al* Mac‐2‐binding protein is a diagnostic marker for biliary tract carcinoma. Cancer 2004; 101: 1609–1615. 15378479 10.1002/cncr.20469

[35] Sundaram M , Zhong S , Bou Khalil M , *et al* Expression of apolipoprotein C‐III in McA‐RH7777 cells enhances VLDL assembly and secretion under lipid‐rich conditions. J Lipid Res 2010; 51: 150–161. 19622837 10.1194/jlr.M900346-JLR200 PMC2789775

[36] Noris M , Remuzzi G . Overview of complement activation and regulation. Semin Nephrol 2013; 33: 479–492. 24161035 10.1016/j.semnephrol.2013.08.001 PMC3820029

[37] Steel LF , Shumpert D , Trotter M , *et al* A strategy for the comparative analysis of serum proteomes for the discovery of biomarkers for hepatocellular carcinoma. Proteomics 2003; 3: 601–609. 12748940 10.1002/pmic.200300399

[38] Zhu C , Song H , Xu F , *et al* Hepatitis B virus inhibits the expression of complement C3 and C4, *in vitro* and *in vivo* . Oncol Lett 2018; 15: 7459–7463. 29731897 10.3892/ol.2018.8223 PMC5920570

[39] Pio R , Corrales L , Lambris JD . The role of complement in tumor growth. Adv Exp Med Biol 2014; 772: 229–262. 24272362 10.1007/978-1-4614-5915-6_11 PMC4379038

[40] Rutkowski MJ , Sughrue ME , Kane AJ , *et al* Cancer and the complement cascade. Mol Cancer Res 2010; 8: 1453–1465. 20870736 10.1158/1541-7786.MCR-10-0225

[41] Stover C . Dual role of complement in tumour growth and metastasis (review). Int J Mol Med 2010; 25: 307–313. 20127033 10.3892/ijmm_00000346

[42] Pio R , Ajona D , Lambris JD . Complement inhibition in cancer therapy. Semin Immunol 2013; 25: 54–64. 23706991 10.1016/j.smim.2013.04.001 PMC3733085

[43] Downs‐Canner S , Magge D , Ravindranathan R , *et al* Complement inhibition: a novel form of immunotherapy for colon cancer. Ann Surg Oncol 2016; 23: 655–662. 26289805 10.1245/s10434-015-4778-7 PMC5733728

[44] Hase S , Kikuchi N , Ikenaka T , *et al* Structures of sugar chains of the third component of human complement. J Biochem 1985; 98: 863–874. 4077844 10.1093/oxfordjournals.jbchem.a135366

[45] Tomana M , Niemann M , Garner C , *et al* Carbohydrate composition of the second, third and fifth components and factors B and D of human complement. Mol Immunol 1985; 22: 107–111. 3844601 10.1016/s0161-5890(85)80004-3

[46] Welinder KG , Svendsen A . Amino acid sequence analysis of the glycopeptides from human complement component C3. FEBS Lett 1986; 202: 59–62. 3087774 10.1016/0014-5793(86)80649-4

[47] Ricklin D , Hajishengallis G , Yang K , *et al* Complement: a key system for immune surveillance and homeostasis. Nat Immunol 2010; 11: 785–797. 20720586 10.1038/ni.1923 PMC2924908

[48] Chou PY , Fasman GD . Prediction of the secondary structure of proteins from their amino acid sequence. Adv Enzymol Relat Areas Mol Biol 1978; 47: 45–148. 364941 10.1002/9780470122921.ch2

