# Supplementary material for: Plasma proteome plus site‐specific N‐glycoprofiling for hepatobiliary carcinomas
Source: J Pathol Clin Res. 2019 Jun 25;5(3):199–212. doi: 10.1002/cjp2.136 (PMC6648390; doi:10.1002/cjp2.136)
Supplement: Supplementary file 1 — Figure S1. Flowchart of the study design Figure S2. Comparisons of plasma proteome/peptidome and N‐glycoproteome/N‐glycopeptidome among HCC, CCA, cHCC‐CCA, and controls. Figure S3. Factors associated with tumor stage and grade Figure S4. Kaplan–Meier analyses of recurrence‐free and overall survival Figure S5. Correlations between protein content and protein concentration of complement C3, apolipoprotein C‐III, and galectin‐3‐binding protein Figure S6. Levels of α‐fetoprotein in different tumor grades [file CJP2-5-199-s001.docx]

**Plasma proteome plus site-specific *N*-glycoprofiling for hepatobiliary carcinomas**

Chang T-T *et al*. *J Pathol Clin Res* DOI: 10.1002/cjp2.136

**Supplementary Figures**


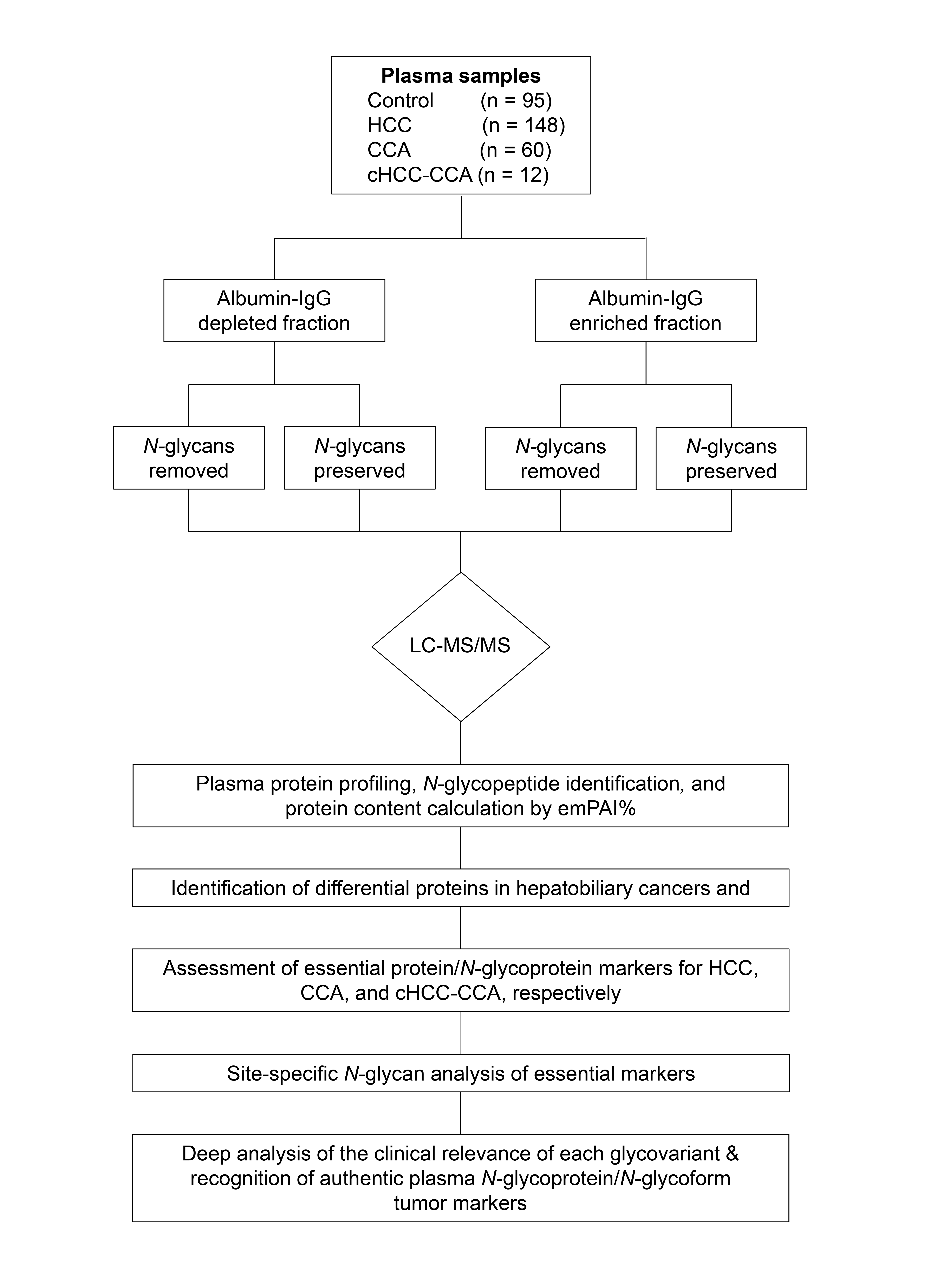


**Figure S1.** Flowchart of the study design. CCA, cholangiocarcinoma; cHCC-CCA, combined hepatocellular cholangiocarcinoma; emPAI, exponentially modified protein abundance index; HCC, hepatocellular carcinoma; LC-MS/MS, Liquid chromatography–tandem mass spectrometry.


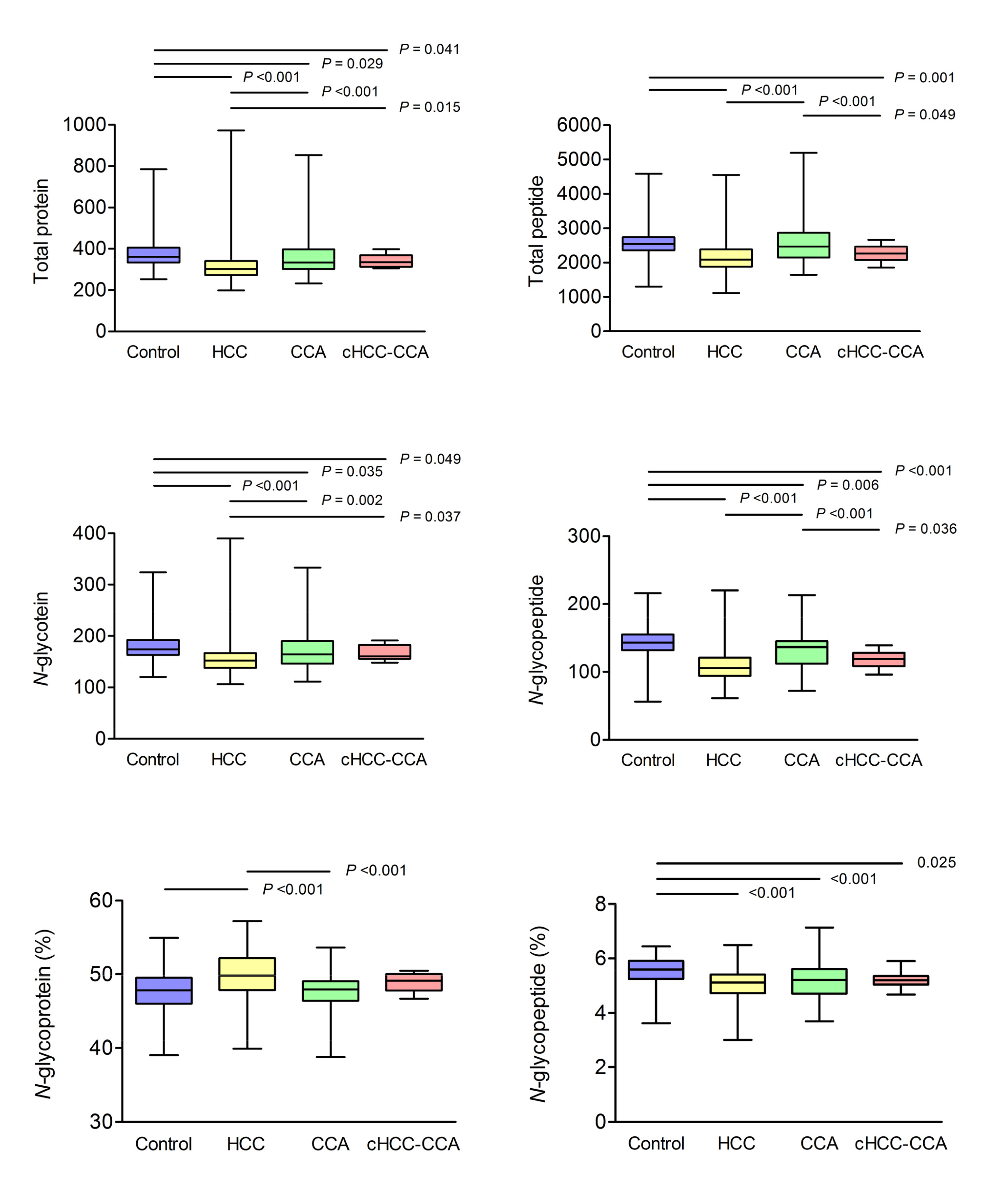


**Figure S2.** Comparisons of plasma proteome/peptidome and *N*-glycoproteome/*N*-glycopeptidome among hepatocellular carcinoma (HCC), cholangiocarcinoma (CCA), combined hepatocellular cholangiocarcinoma (cHCC-CCA), and controls are shown as box-and-whisker plots. *P*-values are obtained from Kruskal-Wallis tests with Dunn's post hoc tests.


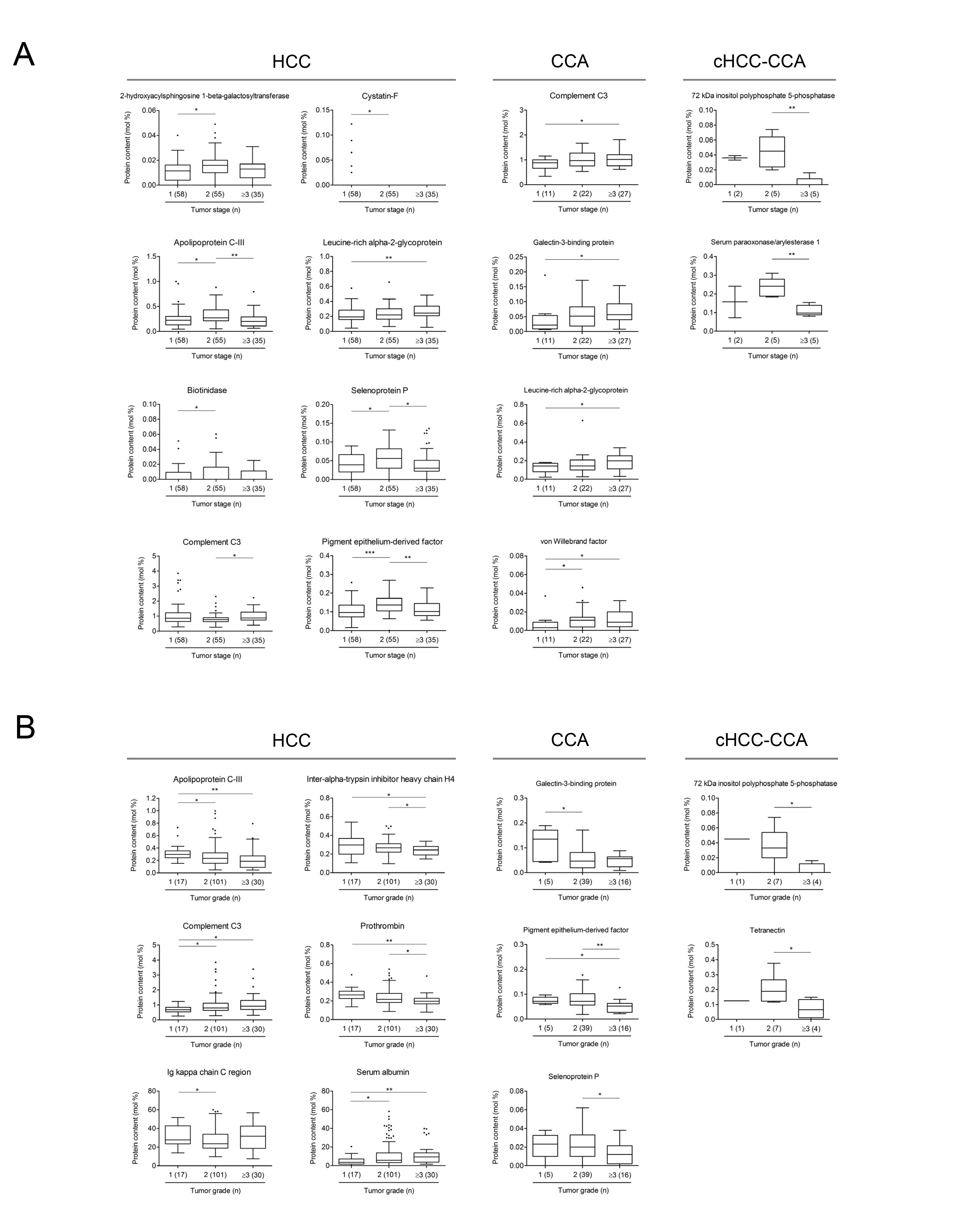


**Figure S3.** Factors associated with (A) tumor stage and (B) tumor grade of hepatocellular carcinoma (HCC; n = 148), cholangiocarcinoma (CCA; n = 60), and combined hepatocellular cholangiocarcinoma (cHCC-CCA; n = 12) are shown as box-and-whisker plots. *P*-values are obtained from Kruskal-Wallis tests with Dunn's post hoc tests (*, *P*<0.05; **, *P*<0.01; ***, *P*<0.001).


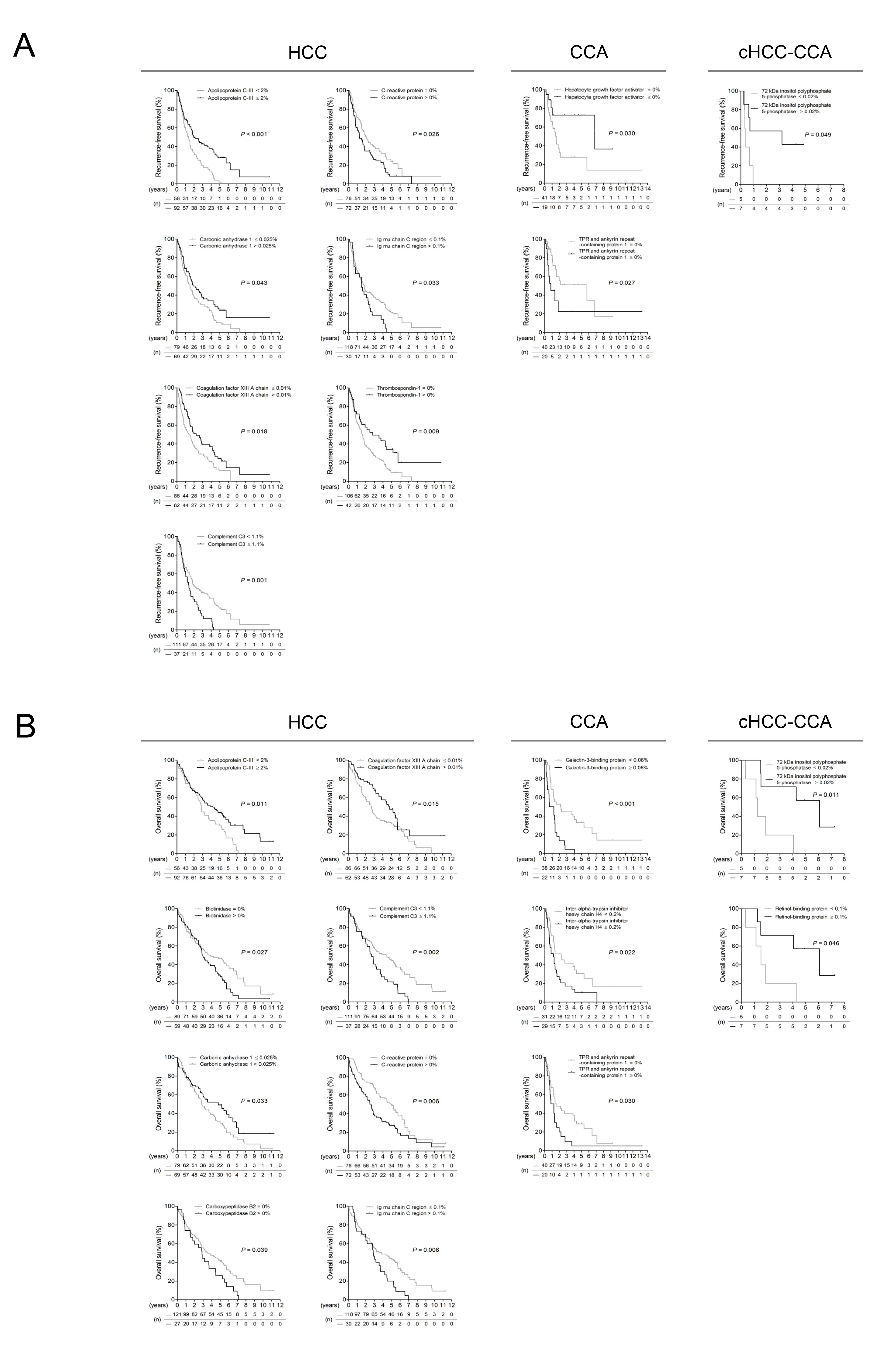


**Figure S4.** Kaplan-Meier analyses of (A) recurrence-free survival and (B) overall survival in 148 patients with hepatocellular carcinoma (HCC), 60 patients with cholangiocarcinoma (CCA), and 12 patients with combined hepatocellular cholangiocarcinoma (cHCC-CCA). *P*-values are obtained from log-rank tests.


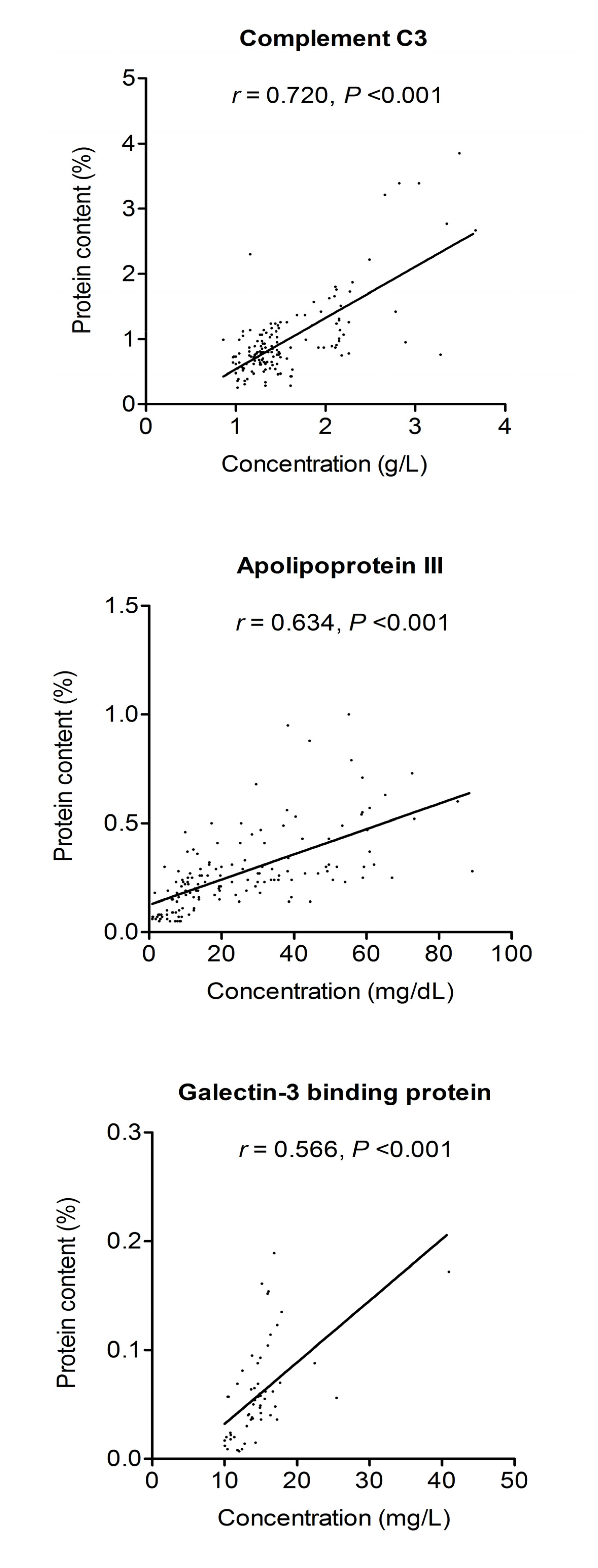


**Figure S5.** Correlations between protein content (evaluated by the percentage of the exponentially modified protein abundance index) and protein concentration of complement C3, apolipoprotein C-III, and galectin-3-binding protein in plasma. The coefficient *r* and *P*-value are obtained from the Pearson correlation tests.


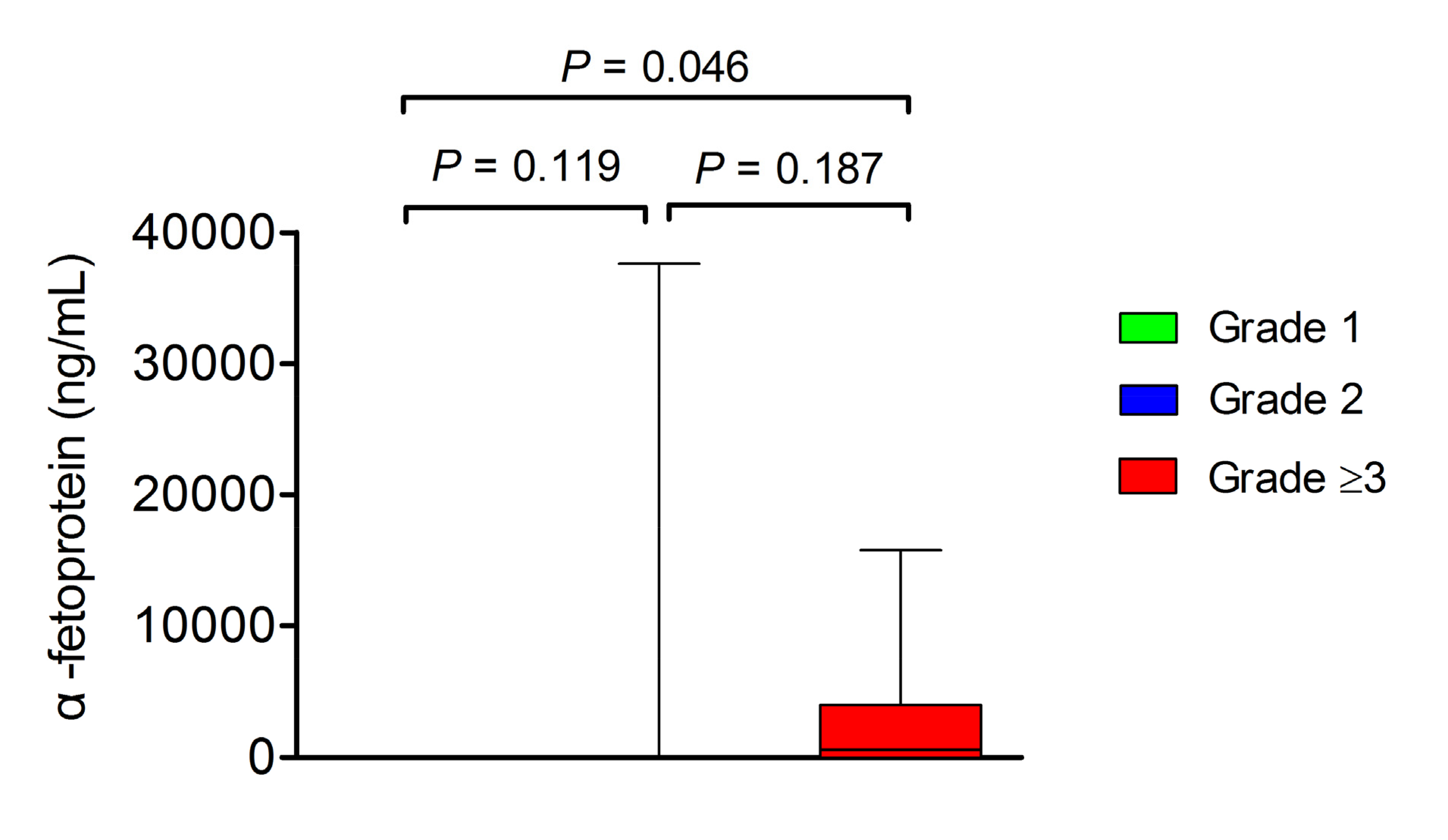


**Figure S6.** Levels of α-fetoprotein in different tumor grades are shown as box-and-whisker plots. *P*-values are obtained from Kruskal-Wallis tests with Dunn's post hoc tests.
