# Supplementary material for: Plasma proteome plus site‐specific N‐glycoprofiling for hepatobiliary carcinomas
Source: J Pathol Clin Res. 2019 Jun 25;5(3):199–212. doi: 10.1002/cjp2.136 (PMC6648390; doi:10.1002/cjp2.136)
Supplement: Supplementary file 2 — Table S1. Comparisons of characteristics of the patients with different types of hepatobiliary cancer [file CJP2-5-199-s002.docx]

**Plasma proteome plus site-specific *N*-glycoprofiling for hepatobiliary carcinomas**

Chang T-T *et al*. *J Pathol Clin Res* DOI: 10.1002/cjp2.136

| **Table S1.** Comparisons of characteristics of the patients with different types of hepatobiliary cancer | | | | | | |
| --- | --- | --- | --- | --- | --- | --- |
| Variable | HCC (n = 148) | CCA (n = 60) | cHCC-CCA (n = 12) | *P*-value 1 | *P*-value 2 | *P*-value 3 |
| **Demographic, Biochemical, and hematological data** |  |  |  |  |  |  |
| Male, n (%) | 110 (74.3) | 31 (51.7) | 9 (75.0) | 0.003 | 0.205 | 1.000 |
| Age (years) | 60.0 (23.0 - 86.0) | 65.5 (33.0 - 85.0) | 59.5 (36.0 - 71.0) | 0.051 | 0.077 | 0.389 |
| ALT (U/L) | 51.5 (10.0 - 436.0) | 36.5 (10.0 - 199.0) | 44.0 (13.0 - 127.0) | < 0.001 | 0.071 | 0.944 |
| AST (U/L) | 52.0 (17.0 - 800.0) | 45.0 (17.0 - 231.0) | 48.0 (28.0 - 205.0) | 0.028 | 0.167 | 0.799 |
| Alk-P (U/L) | 96.0 (46.0 - 976.0) | 138.0 (25.0 - 786.0) | 137.5 (77.0 - 842.0) | 0.002 | 0.782 | 0.095 |
| Albumin (g/dL) | 4.2 (1.8 - 5.1) | 4.2 (2.9 - 5.2) | 4.4 (3.1 - 4.9) | 0.262 | 0.266 | 0.537 |
| Total bilirubin (mg/dL) | 0.6 (0.2 - 7.0) | 0.6 (0.2 - 11.8) | 0.6 (0.2 - 4.4) | 0.436 | 0.693 | 0.272 |
| Creatinine (mg/dL) | 0.9 (0.4 - 10.8) | 0.8 (0.4 - 7.9) | 0.8 (0.6 - 1.0) | < 0.001 | 0.933 | 0.020 |
| White blood cell (10^3^/μL) | 5.7 (2.0 - 10.4) | 7.0 (3.6 - 16.9) | 6.6 (3.5 - 9.5) | < 0.001 | 0.341 | 0.368 |
| Red blood cell (10^6^/μL) | 4.2 (2.4 - 6.1) | 4.2 (2.6 - 5.6) | 4.4 (2.8 - 5.3) | 0.596 | 0.267 | 0.363 |
| Hemoglobin (g/dL) | 13.4 (8.1 - 17.7) | 12.8 (8.8 - 15.5) | 14.4 (8.5 - 16.9) | 0.036 | 0.009 | 0.096 |
| Hematocrit (%) | 38.6 (24.0 - 50.1) | 37.3 (25.6 - 44.7) | 41.4 (25.3 - 50.1) | 0.105 | 0.027 | 0.108 |
| Platelet (10^3^/μL) | 173.0 (33.0 - 549.0) | 215.5 (84.0 - 412.0) | 206.5 (93.0 - 484.0) | < 0.001 | 0.706 | 0.198 |
| **Tumor-related factors** |  |  |  |  |  |  |
| α-fetoprotein (ng/mL) | 38.7 (0.9 - 45128.0) | 3.1 (1.3 - 474.2) | 42.8 (8.1 - 60500.0) | < 0.001 | < 0.001 | 0.877 |
| CEA (ng/mL) | 2.1 (0.4 - 11.1) | 2.9 (0.3 - 60.4) | 3.3 (2.2 - 4.4) | 0.016 | 0.444 | 0.960 |
| CA 19-9 (U/mL) | 20.0 (0.6 - 32770.0) | 220.0 (0.1 - 36622.0) | 112.7 (73.6 - 248.7) | 0.001 | 0.551 | 0.167 |
| Hepatitis B, n (%) | 84 (56.8) | 19 (31.7) | 8 (66.7) | 0.001 | 0.023 | 0.506 |
| Hepatitis C, n (%) | 49 (33.1) | 7 (11.7) | 3 (25.0) | 0.001 | 0.226 | 0.555 |
| Fatty liver, n (%) | 34 (23.0) | NA | 4 (33.3) | NA | NA | 0.481 |
| Liver cirrhosis, n (%) | 90 (60.8) | NA | 2 (16.7) | NA | NA | 0.004 |
| Tumor staging, n (%) |  |  |  | < 0.001 | 0.538 | 0.016 |
| I | 58 (39.2) | 11 (18.3) | 2 (16.7) |  |  |  |
| II | 55 (37.2) | 22 (36.7) | 5 (41.7) |  |  |  |
| III | 31 (20.9) | 6 (10.0) | 3 (25.0) |  |  |  |
| IVA | 2 (1.4) | 18 (30.0) | 2 (16.7) |  |  |  |
| IVB | 2 (1.4) | 3 (5.0) | 0 (0.0) |  |  |  |
| Follow-up period (years) | 3.2 (0.0 - 11.3) | 1.3 (0.1 - 13.2) | 3.0 (0.3 - 7.3) | < 0.001 | 0.108 | 0.693 |
| 5-year recurrence, n (%) | 107 (72.3) | 25 (41.7) | 9 (75.0) | < 0.001 | 0.056 | 0.755 |
| 5-year survivals, n (%) | 58 (39.2) | 13 (21.7) | 4 (33.3) | 0.016 | 0.460 | 0.767 |
| Data are numbers (percentages) or median values (minimum - maximum). For the patients with CCA, 10 are perihilar type and 50 are intrahepatic type.  Nominal values are compared using Fisher’s exact tests or Pearson Chi square tests. Continuous variables are compared using Mann-Whitney *U* tests. *P*-value 1: comparisons between HCC and CCA groups; *P*-value 2: comparisons between CCA and cHCC-CCA groups; *P*-value 3: comparisons between HCC and cHCC-CCA groups.  Abbreviations: Alk-P, alkaline phosphatase; ALT, alanine transaminase; AST, aspartate aminotransferase; CA 19-9, carbohydrate antigen 19-9; CCA, cholangiocarcinoma; CEA, carcinoembryonic antigen; cHCC-CCA, combined hepatocellular carcinoma and cholangiocarcinoma; HCC, hepatocellular carcinoma; NA, not available. | | | | | | |
