# Supplementary material for: Plasma proteome plus site‐specific N‐glycoprofiling for hepatobiliary carcinomas
Source: J Pathol Clin Res. 2019 Jun 25;5(3):199–212. doi: 10.1002/cjp2.136 (PMC6648390; doi:10.1002/cjp2.136)
Supplement: Supplementary file 3 — Table S2. Comparison of differential protein content between patients with hepatobiliary cancers and controls [file CJP2-5-199-s003.docx]

**Plasma proteome plus site-specific *N*-glycoprofiling for hepatobiliary carcinomas**

Chang T-T *et al*. *J Pathol Clin Res* DOI: 10.1002/cjp2.136

| **Table S2.** Comparison of differential protein content by the percentage of exponential modified protein abundance index between patients with hepatobiliary cancers (n = 220) and controls (n = 95) | | | | |
| --- | --- | --- | --- | --- |
| Variable | Controls | Patients with hepatobiliary cancers | Fold change | *P*-value |
| ***Higher in tumor*** |  |  |  |  |
| 2-hydroxyacylsphingosine 1-beta-galactosyltransferase | 0.00 (0.00 – 0.02) | 0.01 (0.00 – 0.05) | 3.86 | <0.001 |
| Apolipoprotein C-III | 0.08 (0.04 – 1.57) | 0.20 (0.00 – 1.00) | 1.75 | <0.001 |
| BPI fold-containing family C protein | 0.00 (0.00 – 0.00) | 0.00 (0.00 – 0.05) | NA | <0.001 |
| Carbonic anhydrase 1 | 0.00 (0.00 – 0.19) | 0.02 (0.00 – 0.52) | 10.29 | <0.001 |
| Coagulation factor XIII A chain | 0.00 (0.00 – 0.03) | 0.01 (0.00 – 0.07) | 27.00 | <0.001 |
| C-reactive protein | 0.00 (0.00 – 0.07) | 0.00 (0.00 – 0.19) | 8.18 | <0.001 |
| Galectin-3-binding protein | 0.04 (0.00 – 0.19) | 0.07 (0.00 – 0.29) | 1.94 | <0.001 |
| Ig heavy chain V-III region KOL | 0.17 (0.00 – 3.08) | 0.87 (0.00 – 10.75) | 5.17 | <0.001 |
| Ig heavy chain V-III region NIE | 0.22 (0.00 – 2.47) | 0.76 (0.00 – 9.63) | 5.11 | <0.001 |
| Ig kappa chain C region | 13.51 (1.05 – 55.62) | 23.09 (0.00 – 63.94) | 1.61 | <0.001 |
| Ig kappa chain V-III region B6 | 0.00 (0.00 – 1.73) | 0.55 (0.00 – 10.16) | 13.25 | <0.001 |
| Ig lambda chain V-I region NEW | 0.00 (0.00 – 1.49) | 0.33 (0.00 – 11.11) | 10.09 | <0.001 |
| Ig lambda chain V-I region NEWM | 0.00 (0.00 – 1.62) | 0.51 (0.00 – 11.48) | 7.07 | <0.001 |
| Ig lambda chain V-II region BOH | 0.00 (0.00 – 1.23) | 0.00 (0.00 – 10.74) | 21.56 | <0.001 |
| Ig lambda chain V-IV region Hil | 0.18 (0.00 – 1.11) | 0.80 (0.00 – 28.57) | 10.07 | <0.001 |
| Ig lambda-2 chain C regions | 1.92 (0.00 – 20.92) | 5.96 (0.00 – 24.14) | 1.96 | <0.001 |
| Inter-alpha-trypsin inhibitor heavy chain H4 | 0.19 (0.07 – 0.37) | 0.25 (0.06 – 0.54) | 1.30 | <0.001 |
| Leucine-rich alpha-2-glycoprotein | 0.14 (0.00 – 0.36) | 0.20 (0.02 – 0.66) | 1.53 | <0.001 |
| Pigment epithelium-derived factor | 0.07 (0.00 – 0.16) | 0.10 (0.00 – 0.27) | 1.52 | <0.001 |
| Selenoprotein P | 0.02 (0.00 – 0.09) | 0.03 (0.00 – 0.14) | 2.06 | <0.001 |
| Sialic acid-binding Ig-like lectin 16 | 0.00 (0.00 – 0.00) | 0.01 (0.00 – 0.05) | NA | <0.001 |
| TPR and ankyrin repeat-containing protein 1 | 0.00 (0.00 – 0.00) | 0.00 (0.00 – 0.01) | 6.75 | <0.001 |
| UDP-glucose:glycoprotein glucosyltransferase 2 | 0.00 (0.00 – 0.01) | 0.00 (0.00 – 0.02) | 7.78 | <0.001 |
| von Willebrand factor | 0.00 (0.00 – 0.02) | 0.01 (0.00 – 0.08) | 3.84 | <0.001 |
|  |  |  |  |  |
| ***Lower in tumor*** |  |  |  |  |
| 72 kDa inositol polyphosphate 5-phosphatase | 0.02 (0.00 – 0.10) | 0.00 (0.00 – 0.07) | 0.15 | <0.001 |
| Ankyrin repeat and sterile alpha motif domain-containing protein 1B | 0.00 (0.00 – 0.03) | 0.00 (0.00 – 0.01) | 0.09 | <0.001 |
| Apolipoprotein A-I | 14.60 (0.20 – 41.81) | 7.33 (1.22 – 29.24) | 0.58 | <0.001 |
| Biotinidase | 0.01 (0.00 – 0.07) | 0.00 (0.00 – 0.06) | 0.34 | <0.001 |
| Carboxypeptidase B2 | 0.02 (0.00 – 0.06) | 0.00 (0.00 – 0.05) | 0.20 | <0.001 |
| Complement C3 | 1.12 (0.66 – 2.23) | 0.87 (0.26 – 3.85) | 0.82 | <0.001 |
| Cystatin-F | 0.03 (0.00 – 0.20) | 0.00 (0.00 – 0.67) | 0.32 | <0.001 |
| Dynein heavy chain domain-containing protein 1 | 0.00 (0.00 – 0.01) | 0.00 (0.00 – 0.02) | 0.19 | <0.001 |
| Hepatocyte growth factor activator | 0.01 (0.00 – 0.05) | 0.00 (0.00 – 0.14) | 0.42 | <0.001 |
| Ig lambda chain V region 4A | 0.08 (0.00 – 0.57) | 0.00 (0.00 – 0.86) | 0.60 | <0.001 |
| Ig mu chain C region | 0.33 (0.00 – 1.36) | 0.00 (0.00 – 2.78) | 0.37 | <0.001 |
| Insulin-like growth factor-binding protein complex acid labile subunit | 0.04 (0.00 – 0.11) | 0.01 (0.00 – 0.08) | 0.49 | <0.001 |
| Kinesin heavy chain isoform 5C | 0.01 (0.00 – 0.04) | 0.00 (0.00 – 0.04) | 0.24 | <0.001 |
| Kinesin-like protein KIF13B | 0.00 (0.00 – 0.02) | 0.00 (0.00 – 0.04) | 0.24 | <0.001 |
| *N*-acetylmuramoyl-L-alanine amidase | 0.06 (0.00 – 0.15) | 0.04 (0.00 – 0.12) | 0.61 | <0.001 |
| Pericentriolar material 1 protein | 0.00 (0.00 – 0.05) | 0.00 (0.00 – 0.06) | 0.60 | <0.001 |
| Phosphatidylinositol-glycan-specific phospholipase D | 0.01 (0.00 – 0.04) | 0.00 (0.00 – 0.06) | 0.51 | <0.001 |
| Platelet basic protein | 0.28 (0.00 – 1.03) | 0.04 (0.00 – 0.41) | 0.21 | <0.001 |
| Platelet factor 4 | 0.14 (0.00 – 0.49) | 0.00 (0.00 – 0.41) | 0.07 | <0.001 |
| Protein MENT | 0.01 (0.00 – 0.08) | 0.00 (0.00 – 0.05) | 0.25 | <0.001 |
| Prothrombin | 0.41 (0.06 – 0.83) | 0.22 (0.08 – 0.54) | 0.62 | <0.001 |
| Pseudouridylate synthase 7 homolog-like protein | 0.01 (0.00 – 0.06) | 0.00 (0.00 – 0.08) | 0.24 | <0.001 |
| Retinol-binding protein 4 | 0.33 (0.00 – 0.91) | 0.19 (0.00 – 0.95) | 0.60 | <0.001 |
| Serotransferrin | 6.68 (2.85 – 12.79) | 4.63 (1.05 – 11.02) | 0.72 | <0.001 |
| Serum albumin | 56.53 (6.78 – 88.76) | 11.01 (0.00 – 91.00) | 0.37 | <0.001 |
| Serum paraoxonase/arylesterase 1 | 0.28 (0.00 – 0.53) | 0.10 (0.00 – 0.48) | 0.43 | <0.001 |
| Spectrin beta chain, non-erythrocytic 4 | 0.00 (0.00 – 0.02) | 0.00 (0.00 – 0.03) | 0.32 | <0.001 |
| Tetranectin | 0.11 (0.00 – 0.40) | 0.05 (0.00 – 0.43) | 0.48 | <0.001 |
| THAP domain-containing protein 4 | 0.03 (0.00 – 0.18) | 0.00 (0.00 – 0.12) | 0.27 | <0.001 |
| Thrombospondin-1 | 0.04 (0.00 – 0.10) | 0.00 (0.00 – 0.07) | 0.16 | <0.001 |
| Thymosin beta-4 | 0.11 (0.00 – 2.27) | 0.00 (0.00 – 0.82) | 0.06 | <0.001 |
| Trinucleotide repeat-containing gene 6C protein | 0.00 (0.00 – 0.02) | 0.00 (0.00 – 0.03) | 0.47 | <0.001 |
| Vasodilator-stimulated phosphoprotein | 0.00 (0.00 – 0.08) | 0.00 (0.00 – 0.02) | 0.01 | <0.001 |
| Data are median values (minimum - maximum). Variables are compared using Mann-Whitney *U* tests. The fold change is calculated by the mean value of the protein content in the patients divided by that in the controls. NA, not available because proteins are not detected in controls | | | | |
