# Supplementary material for: Plasma proteome plus site‐specific N‐glycoprofiling for hepatobiliary carcinomas
Source: J Pathol Clin Res. 2019 Jun 25;5(3):199–212. doi: 10.1002/cjp2.136 (PMC6648390; doi:10.1002/cjp2.136)
Supplement: Supplementary file 4 — Table S3. Changes in mRNA levels of differential proteins in hepatobiliary cancers [file CJP2-5-199-s004.docx]

**Plasma proteome plus site-specific *N*-glycoprofiling for hepatobiliary carcinomas**

Chang T-T *et al*. *J Pathol Clin Res* DOI: 10.1002/cjp2.136

| **Table S3.** Changes in mRNA levels of differential proteins in hepatobiliary cancers | | | | | |
| --- | --- | --- | --- | --- | --- |
| Variable | Chen et al. (1) | Mas et al. (2) | Roessler et al. (3) | Roessler et al. (4) | Wurmbach et al. (5) |
| n (cancer : normal) | Not consistent | 38 : 19 | 22 : 21 | 225 : 220 | 35 : 10 |
| ***Higher in tumor*** |  |  |  |  |  |
| 2-hydroxyacylsphingosine 1-beta-galactosyltransferase | -1.110 (0.782) | -1.027 (0.895) | -1.027 (0.798) | 1.018 (0.156) | 1.024 (0.280) |
| Apolipoprotein C-III | -1.780 (1.000) | NA | -2.935 (0.999) | -2.038 (1.000) | -1.042 (1.000) |
| BPI fold-containing family C protein | NA | NA | NA | NA | NA |
| Carbonic anhydrase 1 | 1.051 (0.272) | -1.256 (1.000) | -1.087 (0.852) | -1.021 (0.961) | NA |
| Coagulation factor XIII A chain | 1.450 (3.7E-5) | 2.122 (7.9E-6) | 1.080 (0.178) | 1.304 (3.4E-9) | 2.65 (0.011) |
| C-reactive protein | NA | -5.376 (1.000) | -1.163 (0.606) | -1.702 (1.000) | -4.233 (0.992) |
| Galectin-3-binding protein | -1.003 (0.509) | 3.420 (8.4E-10) | 1.109 (0.360) | -1.211 (0.994) | 3.427 (6.7E-5) |
| Ig heavy chain V-III region KOL | NA | NA | NA | NA | NA |
| Ig heavy chain V-III region NIE | NA | NA | NA | NA | NA |
| Ig kappa chain C region | 1.286 (6.2E-4) | 5.582 (2.3E-13) | -1.426 (0.983) | 1.025 (0.021) | 1.722 (0.007) |
| Ig kappa chain V-III region B6 | NA | NA | NA | NA | NA |
| Ig lambda chain V-I region NEW | NA | NA | NA | NA | NA |
| Ig lambda chain V-I region NEWM | NA | NA | NA | NA | NA |
| Ig lambda chain V-II region BOH | NA | NA | NA | NA | NA |
| Ig lambda chain V-IV region Hil | NA | NA | NA | NA | NA |
| Ig lambda-2 chain C regions | NA | NA | NA | NA | NA |
| Inter-alpha-trypsin inhibitor heavy chain H4 | -2.554 (1.000) | -1.930 (1.000) | -5.573 (1.000) | -3.839 (1.000) | 1.225 (0.138) |
| Leucine-rich alpha-2-glycoprotein | NA | NA | NA | NA | -4.361 (1.000) |
| Pigment epithelium-derived factor | NA | -1.091 (0.918) | -1.555 (0.999) | -1.522 (1.000) | -1.418 (0.994) |
| Selenoprotein P | -1.110 (0.962) | -1.060 (0.901) | -1.468 (1.000) | -1.356 (1.000) | NA |
| Sialic acid-binding Ig-like lectin 16 | NA | NA | NA | NA | -1.249 (0.966) |
| TPR and ankyrin repeat-containing protein 1 | -1.269 (1.000) | NA | -1.303 (0.999) | -1.579 (1.000) | -1.182 (0.823) |
| UDP-glucose:glycoprotein glucosyltransferase 2 | 1.044 (0.283) | -1.130 (0.986) | 1.235 (0.006) | 1.203 (6.8E-10) | 1.305 (0.002) |
| von Willebrand factor | 1.878 (1.7E-7) | 2.803 (2.7E-10) | 1.766 (0.002) | 1.543 (1.1E-12) | 6.649 (8.3E-7) |
|  |  |  |  |  |  |
| ***Lower in tumor*** |  |  |  |  |  |
| 72 kDa inositol polyphosphate 5-phosphatase | -1.455 (4.7E-13) | -1.029 (0.291) | 1.267 (0.987) | -1.076 (0.007) | 1.257 (0.976) |
| Ankyrin repeat and sterile alpha motif domain-containing protein 1B | 1.313 (0.983) | 1.009 (0.743) | -1.027 (0.069) | -1.016 (0.099) | NA |
| Apolipoprotein A-I | -5.461 (3.8E-19) | -1.239 (0.027) | -2.050 (0.001) | -2.447 (3.2E-25) | -1.919 (5.7E-10) |
| Biotinidase | -1.291 (0.001) | 1.194 (0.969) | -1.531 (6.1E-4) | -1.227 (6.0E-6) | -1.843 (5.2E-4) |
| Carboxypeptidase B2 | -2.368 (2.9E-15) | -1.234 (3.2E-4) | -2.864 (4.4E-9) | -1.861 (1.0E-20) | -1.717 (2.0E-6) |
| Complement C3 | NA | -1.308 (1.4E-5) | -1.468 (1.3E-5) | -1.461 (1.8E-17) | -1.308 (0.015) |
| Cystatin-F | -1.732 (1.7E-6) | 1.237 (1.000) | -1.188 (0.003) | -1.236 (8.8E-10) | 1.067 (0.584) |
| Dynein heavy chain domain-containing protein 1 | -1.354 (0.003) | NA | NA | NA | -1.093 (0.008) |
| Hepatocyte growth factor activator | NA | -1.397 (0.014) | -9.059 (7.9E-10) | -8.182 (5.9E-76) | -6.182 (1.1E-6) |
| Ig lambda chain V region 4A | NA | NA | NA | NA | NA |
| Ig mu chain C region | NA | 2.964 (1.000) | -4.857 (1.3E-8) | -3.657 (3.6E-39) | -1.689 (0.060) |
| Insulin-like growth factor-binding protein complex acid labile subunit | NA | -1.895 (1.1E-9) | -3.881 (3.3E-12) | -4.110 (1.8E-88) | -8.107 (8.1E-10) |
| Kinesin heavy chain isoform 5C | -1.239 (0.002) | -1.014 (0.358) | 1.064 (0.908) | 1.044 (0.972) | NA |
| Kinesin-like protein KIF13B | -1.069 (0.164) | -1.166 (0.004) | -1.244 (0.059) | -1.293 (1.7E-11) | -1.231 (0.041) |
| *N*-acetylmuramoyl-L-alanine amidase | NA | NA | NA | NA | -6.955 (9.6E-7) |
| Pericentriolar material 1 protein | 1.035 (0.679) | -1.216 (0.002) | -1.042 (0.230) | -1.054 (0.021) | -1.000 (0.499) |
| Phosphatidylinositol-glycan-specific phospholipase D | NA | -1.974 (2.9E-5) | -1.248 (0.062) | -2.030 (3.8E-30) | -2.504 (0.007) |
| Platelet basic protein | -1.274 (1.5E-5) | -1.347 (5.7E-4) | -1.282 (2.4E-5) | -1.199 (4.0E-15) | -2.055 (0.011) |
| Platelet factor 4 | -1.510 (6.6E-6) | -1.219 (9.3E-5) | -1.277 (1.7E-5) | -1.171 (5.5E-11) | -1.336 (0.011) |
| Protein MENT | NA | NA | NA | NA | NA |
| Prothrombin | -2.246 (7.3E-14) | -1.583 (4.6E-70 | -2.049 (9.0E-4) | -1.659 (7.9E-19) | -1.862 (6.5E-4) |
| Pseudouridylate synthase 7 homolog-like protein | NA | -1.180 (1.4E-4) | 1.117 (0.983) | 1.138 (1.000) | 1.153 (0.846) |
| Retinol-binding protein 4 | -2.590 (2.3E-15) | -1.379 (2.3E-5) | -2.117 (2.7E-13) | -1.507 (3.2E-28) | -1.488 (0.015) |
| Serotransferrin | -2.496 (3.6E-16) | 1.018 (0.638) | -2.607 (3.4E-4) | -2.375 (1.0E-29) | -1.232 (0.031) |
| Serum albumin | -3.491 (1.0E-10) | -2.667 (1.0E-8) | -2.320 (2.5E-4) | -3.380 (1.1E-40) | -1.277 (3.2E-5) |
| Serum paraoxonase/arylesterase 1 | -2.392 (6.2E-11) | -1.323 (0.001) | -3.318 (1.0E-9) | -2.221 (7.1E-48) | -4.481 (5.7E-6) |
| Spectrin beta chain, non-erythrocytic 4 | NA | -1.165 (8.4E-4) | -1.317 (0.002) | -1.077 (7.6E-5) | -1.089 (0.011) |
| Tetranectin | -3.035 (7.7E-20) | -1.264 (0.023) | -2.513 (5.6E-7) | -1.937 (1.9E-31) | -2.739 (7.6E-7) |
| THAP domain-containing protein 4 | NA | 1.044 (0.775) | 1.073 (0.812) | -1.038 (0.113) | -1.318 (0.008) |
| Thrombospondin-1 | -5.272 (4.7E-29) | -1.067 (0.027) | -1.189 (2.8E-5) | -1.398 (3.6E-15) | -3.072 (5.2E-5) |
| Thymosin beta-4 | -1.059 (0.253) | NA | NA | NA | NA |
| Trinucleotide repeat-containing gene 6C protein | 1.123 (0.937) | NA | NA | NA | NA |
| Vasodilator-stimulated phosphoprotein | -1.004 (0.475) | -1.186 (0.032) | 1.494 (0.999) | 1.068 (0.962) | 1.089 (0.742) |
| Data are obtained from ONCOMINE and are shown as fold change of the gene expression level in hepatocellular carcinoma tissues relative to the normal liver tissues (*P*-value). Reference: (1) Mol Biol Cell. 2002 Jun;13(6):1929-39; (2) Mol Med. 2009 Mar-Apr;15(3-4):85-94; (3) Cancer Res. 2010 Dec 15;70(24):10202-12; (4) Cancer Res. 2010 Dec 15;70(24):10202-12; (5) Hepatology. 2007 Apr;45(4):938-47. NA, not available. | | | | | |
