# Supplementary material for: Plasma proteome plus site‐specific N‐glycoprofiling for hepatobiliary carcinomas
Source: J Pathol Clin Res. 2019 Jun 25;5(3):199–212. doi: 10.1002/cjp2.136 (PMC6648390; doi:10.1002/cjp2.136)
Supplement: Supplementary file 5 — Table S4. Comparison of differential protein content among different hepatobiliary cancers [file CJP2-5-199-s005.docx]

**Plasma proteome plus site-specific *N*-glycoprofiling for hepatobiliary carcinomas**

Chang T-T *et al*. *J Pathol Clin Res* DOI: 10.1002/cjp2.136

| **Table S4.** Comparison of differential protein content by the percentage of exponential modified protein abundance index among different hepatobiliary cancers | | | | | | |
| --- | --- | --- | --- | --- | --- | --- |
| Variable | HCC (n = 148) | CCA (n = 60) | cHCC-CCA (n = 12) | *P*-value 1 | *P*-value 2 | *P*-value 3 |
| ***Higher in hepatobiliary cancers*** |  |  |  |  |  |  |
| 2-hydroxyacylsphingosine 1-beta-galactosyltransferase | 0.01 (0.00 - 0.05) | 0.01 (0.00 - 0.02) | 0.01 (0.00 - 0.03) | < 0.001 | < 0.001 | 1.000 |
| Apolipoprotein C-III | 0.24 (0.05 – 1.00) | 0.12 (0.00 - 0.44) | 0.20 (0.06 - 0.42) | < 0.001 | 0.034 | 0.325 |
| BPI fold-containing family C protein | 0.00 (0.00 - 0.05) | 0.00 (0.00 - 0.05) | 0.01 (0.00 - 0.03) | < 0.001 | 0.010 | 0.474 |
| Carbonic anhydrase 1 | 0.02 (0.00 - 0.52) | 0.02 (0.00 - 0.33) | 0.01 (0.00 - 0.24) | 0.708 | 0.627 | 0.897 |
| Coagulation factor XIII A chain | 0.01 (0.00 - 0.06) | 0.00 (0.00 - 0.07) | 0.01 (0.00 - 0.05) | 0.013 | 0.032 | 0.311 |
| C-reactive protein | 0.00 (0.00 - 0.19) | 0.00 (0.00 - 0.11) | 0.00 (0.00 - 0.08) | 0.484 | 0.645 | 0.488 |
| Galectin-3-binding protein | 0.08 (0.00 - 0.25) | 0.05 (0.00 - 0.17) | 0.10 (0.02 - 0.29) | < 0.001 | 0.012 | 0.646 |
| Ig heavy chain V-III region KOL | 1.83 (0.00 - 10.75)^a^ | 0.23 (0.00 - 4.51) | 0.32 (0.06 - 0.46) | < 0.001 | 0.198 | 0.002 |
| Ig heavy chain V-III region NIE | 1.46 (0.00 - 9.63)^a^ | 0.27 (0.00 - 2.84) | 0.34 (0.06 - 0.82) | < 0.001 | 0.276 | 0.002 |
| Ig kappa chain C region | 24.79 (7.45 - 60.08)^a^ | 16.57 (0.00 - 63.94) | 12.43 (3.00 - 22.64) | < 0.001 | 0.011 | < 0.001 |
| Ig kappa chain V-III region B6 | 2.39 (0.00 - 10.16)^a^ | 0.25 (0.00 - 3.44) | 0.00 (0.00 - 1.48) | < 0.001 | 0.032 | < 0.001 |
| Ig lambda chain V-I region NEW | 1.05 (0.00 - 11.11)^a^ | 0.07 (0.00 - 2.33) | 0.08 (0.00 - 0.25) | < 0.001 | 0.767 | < 0.001 |
| Ig lambda chain V-I region NEWM | 1.10 (0.00 - 11.48)^a^ | 0.14 (0.00 - 5.67) | 0.31 (0.00 - 0.61) | < 0.001 | 0.074 | 0.020 |
| Ig lambda chain V-II region BOH | 0.98 (0.00 - 10.74)^a^ | 0.00 (0.00 - 2.25) | 0.00 (0.00 – 0.00) | < 0.001 | 0.432 | < 0.001 |
| Ig lambda chain V-IV region Hil | 2.35 (0.00 - 28.57)^a^ | 0.24 (0.00 - 1.32) | 0.51 (0.03 - 1.36) | < 0.001 | 0.055 | 0.003 |
| Ig lambda-2 chain C regions | 7.37 (0.00 - 24.14)^a^ | 3.02 (0.60 - 22.08) | 3.47 (1.07 - 4.46) | < 0.001 | 0.618 | < 0.001 |
| Inter-alpha-trypsin inhibitor heavy chain H4 | 0.26 (0.10 - 0.54) | 0.20 (0.06 - 0.43) | 0.27 (0.10 - 0.37) | < 0.001 | 0.005 | 0.741 |
| Leucine-rich alpha-2-glycoprotein | 0.22 (0.04 - 0.66) | 0.17 (0.02 - 0.63) | 0.18 (0.05 - 0.45) | < 0.001 | 0.586 | 0.117 |
| Pigment epithelium-derived factor | 0.11 (0.00 - 0.27)^a^ | 0.06 (0.02 - 0.18) | 0.11 (0.05 - 0.13) | < 0.001 | 0.002 | 0.389 |
| Selenoprotein P | 0.05 (0.00 - 0.14)^a^ | 0.02 (0.00 - 0.06) | 0.04 (0.02 - 0.08) | < 0.001 | < 0.001 | 0.866 |
| Sialic acid-binding Ig-like lectin 16 | 0.01 (0.00 - 0.05) | 0.00 (0.00 - 0.03) | 0.00 (0.00 - 0.03) | 0.002 | 0.767 | 0.324 |
| TPR and ankyrin repeat-containing protein 1 | 0.00 (0.00 - 0.01) | 0.00 (0.00 - 0.00) | 0.00 (0.00 - 0.01) | < 0.001 | 0.715 | 0.036 |
| UDP-glucose:glycoprotein glucosyltransferase 2 | 0.00 (0.00 - 0.02)^a^ | 0.00 (0.00 - 0.01) | 0.00 (0.00 – 0.00) | < 0.001 | 0.194 | < 0.001 |
| von Willebrand factor | 0.01 (0.00 - 0.06) | 0.01 (0.00 - 0.05) | 0.01 (0.00 - 0.08) | 0.288 | 0.586 | 0.375 |
|  |  |  |  |  |  |  |
| ***Lower in hepatobiliary cancers*** |  |  |  |  |  |  |
| 72 kDa inositol polyphosphate 5-phosphatase | 0.00 (0.00 - 0.00)^b^ | 0.00 (0.00 - 0.06) | 0.02 (0.00 - 0.07) | < 0.001 | < 0.001 | < 0.001 |
| Ankyrin repeat and sterile alpha motif domain-containing protein 1B | 0.00 (0.00 - 0.00)^b^ | 0.00 (0.00 - 0.01) | 0.00 (0.00 - 0.01) | < 0.001 | 0.981 | < 0.001 |
| Apolipoprotein A-I | 7.33 (1.39 - 27.83) | 7.84 (1.22 - 29.24) | 6.81 (4.33 - 13.19) | 0.425 | 0.576 | 0.741 |
| Biotinidase | 0.00 (0.00 - 0.06) | 0.00 (0.00 - 0.03) | 0.00 (0.00 - 0.03) | 0.160 | 0.808 | 0.509 |
| Carboxypeptidase B2 | 0.00 (0.00 - 0.04) | 0.00 (0.00 - 0.05) | 0.00 (0.00 - 0.03) | < 0.001 | 0.128 | 0.962 |
| Complement C3 | 0.80 (0.26 - 3.85) | 0.96 (0.34 - 1.82) | 1.02 (0.56 - 1.43) | 0.028 | 0.406 | 0.064 |
| Cystatin-F | 0.00 (0.00 - 0.12)^b^ | 0.00 (0.00 - 0.67) | 0.00 (0.00 - 0.18) | < 0.001 | 0.055 | < 0.001 |
| Dynein heavy chain domain-containing protein 1 | 0.00 (0.00 - 0.02) | 0.00 (0.00 - 0.00) | 0.00 (0.00 - 0.01) | 0.580 | 0.002 | < 0.001 |
| Hepatocyte growth factor activator | 0.00 (0.00 - 0.14) | 0.00 (0.00 - 0.02) | 0.00 (0.00 - 0.01) | 0.553 | 0.875 | 0.628 |
| Ig lambda chain V region 4A | 0.00 (0.00 - 0.76)^b^ | 0.10 (0.00 - 0.86) | 0.16 (0.00 - 0.23) | < 0.001 | 0.042 | < 0.001 |
| Ig mu chain C region | 0.00 (0.00 - 2.78) | 0.22 (0.00 - 0.60) | 0.00 (0.00 - 0.73) | < 0.001 | 0.508 | 0.091 |
| Insulin-like growth factor-binding protein complex acid labile subunit | 0.01 (0.00 - 0.08) | 0.01 (0.00 - 0.05) | 0.02 (0.00 - 0.04) | 0.943 | 0.794 | 0.849 |
| Kinesin heavy chain isoform 5C | 0.00 (0.00 - 0.00)^b^ | 0.00 (0.00 - 0.03) | 0.02 (0.00 - 0.04) | < 0.001 | < 0.001 | < 0.001 |
| Kinesin-like protein KIF13B | 0.00 (0.00 - 0.04) | 0.00 (0.00 - 0.01) | 0.01 (0.00 - 0.02) | 0.004 | < 0.001 | < 0.001 |
| *N*-acetylmuramoyl-L-alanine amidase | 0.04 (0.00 - 0.12) | 0.04 (0.00 - 0.09) | 0.06 (0.02 - 0.10) | 0.943 | 0.015 | 0.011 |
| Pericentriolar material 1 protein | 0.00 (0.00 - 0.06) | 0.00 (0.00 - 0.03) | 0.01 (0.00 - 0.01) | 0.438 | < 0.001 | < 0.001 |
| Phosphatidylinositol-glycan-specific phospholipase D | 0.00 (0.00 - 0.06) | 0.00 (0.00 - 0.03) | 0.00 (0.00 - 0.02) | 0.342 | 0.315 | 0.173 |
| Platelet basic protein | 0.04 (0.00 - 0.37) | 0.04 (0.00 - 0.41) | 0.06 (0.00 - 0.41) | 0.409 | 0.516 | 0.279 |
| Platelet factor 4 | 0.00 (0.00 - 0.05)^b^ | 0.00 (0.00 - 0.41) | 0.00 (0.00 - 0.09) | < 0.001 | 0.865 | < 0.001 |
| Protein MENT | 0.00 (0.00 - 0.05) | 0.00 (0.00 - 0.03) | 0.00 (0.00 - 0.03) | 0.030 | 0.012 | 0.265 |
| Prothrombin | 0.22 (0.08 - 0.54) | 0.23 (0.08 - 0.54) | 0.33 (0.17 - 0.41) | 0.656 | 0.030 | 0.004 |
| Pseudouridylate synthase 7 homolog-like protein | 0.00 (0.00 - 0.08) | 0.00 (0.00 - 0.05) | 0.00 (0.00 - 0.02) | < 0.001 | 0.083 | 0.949 |
| Retinol-binding protein 4 | 0.21 (0.00 - 0.95) | 0.15 (0.00 - 0.74) | 0.11 (0.00 - 0.38) | 0.036 | 0.506 | 0.090 |
| Serotransferrin | 5.02 (1.49 - 11.02) | 4.30 (1.05 - 7.76) | 3.64 (1.89 - 6.15) | < 0.001 | 0.319 | < 0.001 |
| Serum albumin | 5.72 (0.00 - 58.07)^b^ | 34.78 (2.20 - 80.78) | 43.72 (19.67 - 91.00) | < 0.001 | 0.026 | < 0.001 |
| Serum paraoxonase/arylesterase 1 | 0.10 (0.00 - 0.48) | 0.09 (0.00 - 0.29) | 0.17 (0.07 - 0.31) | 0.414 | 0.007 | 0.008 |
| Spectrin beta chain, non-erythrocytic 4 | 0.00 (0.00 - 0.03) | 0.00 (0.00 - 0.01) | 0.01 (0.00 - 0.02) | 0.024 | < 0.001 | < 0.001 |
| Tetranectin | 0.05 (0.00 - 0.43) | 0.03 (0.00 - 0.27) | 0.14 (0.00 - 0.38) | 0.113 | < 0.001 | < 0.001 |
| THAP domain-containing protein 4 | 0.00 (0.00 - 0.00)^b^ | 0.00 (0.00 - 0.12) | 0.08 (0.02 - 0.10) | < 0.001 | < 0.001 | < 0.001 |
| Thrombospondin-1 | 0.00 (0.00 - 0.07) | 0.00 (0.00 - 0.05) | 0.00 (0.00 - 0.06) | 0.278 | 0.499 | 0.883 |
| Thymosin beta-4 | 0.00 (0.00 - 0.82) | 0.00 (0.00 - 0.28) | 0.00 (0.00 - 0.29) | 0.608 | 0.937 | 0.740 |
| Trinucleotide repeat-containing gene 6C protein | 0.00 (0.00 - 0.01)^b^ | 0.00 (0.00 - 0.03) | 0.01 (0.00 - 0.02) | < 0.001 | 0.127 | < 0.001 |
| Vasodilator-stimulated phosphoprotein | 0.00 (0.00 – 0.00) | 0.00 (0.00 - 0.02) | 0.00 (0.00 – 0.00) | 0.026 | 0.524 | 1.000 |
| Data are median values (minimum - maximum). Variables are compared using Mann-Whitney *U* tests. *P*-value 1: comparisons between HCC and CCA groups; *P*-value 2: comparisons between CCA and cHCC-CCA groups; *P*-value 3: comparisons between HCC and cHCC-CCA groups. ^a^the highest in HCC, ^b^the lowest in HCC. Abbreviations: CCA, cholangiocarcinoma; cHCC-CCA, combined hepatocellular carcinoma and cholangiocarcinoma; HCC, hepatocellular carcinoma. | | | | | | |
