# Supplementary material for: Plasma proteome plus site‐specific N‐glycoprofiling for hepatobiliary carcinomas
Source: J Pathol Clin Res. 2019 Jun 25;5(3):199–212. doi: 10.1002/cjp2.136 (PMC6648390; doi:10.1002/cjp2.136)
Supplement: Supplementary file 6 — Table S5. Comparison of differential protein content between patients with intrahepatic CCA and patients with perihilar CCA [file CJP2-5-199-s006.docx]

**Plasma proteome plus site-specific *N*-glycoprofiling for hepatobiliary carcinomas**

Chang T-T *et al*. *J Pathol Clin Res* DOI: 10.1002/cjp2.136

| **Table S5.** Comparison of differential protein content by the percentage of exponential modified protein abundance index between patients with intrahepatic CCA (n = 50) and patients with perihilar CCA (n = 10) | | | |
| --- | --- | --- | --- |
| Variable | Intrahepatic CCA | Perihilar CCA | *P*-value |
| ***Higher in tumor*** |  |  |  |
| 2-hydroxyacylsphingosine 1-beta-galactosyltransferase | 0.01 (0.00 - 0.02) | 0.01 (0.00 - 0.01) | 0.188 |
| Apolipoprotein C-III | 0.13 (0.00 - 0.44) | 0.08 (0.00 - 0.27) | 0.033 |
| BPI fold-containing family C protein | 0.01 (0.00 - 0.02) | 0.00 (0.00 - 0.01) | 0.162 |
| Carbonic anhydrase 1 | 0.02 (0.00 - 0.33) | 0.02 (0.00 - 0.30) | 0.943 |
| Coagulation factor XIII A chain | 0.00 (0.00 - 0.07) | 0.00 (0.00 - 0.02) | 0.825 |
| C-reactive protein | 0.01 (0.00 - 0.11) | 0.00 (0.00 - 0.07) | 0.623 |
| Galectin-3-binding protein | 0.05 (0.00 - 0.19) | 0.06 (0.01 - 0.16) | 0.153 |
| Ig heavy chain V-III region KOL | 0.19 (0.00 - 4.51) | 0.25 (0.00 - 4.16) | 0.462 |
| Ig heavy chain V-III region NIE | 0.20 (0.00 - 2.84) | 0.40 (0.00 - 2.67) | 0.204 |
| Ig kappa chain C region | 16.50 (0.00 - 63.94) | 17.93 (6.89 - 31.21) | 0.634 |
| Ig kappa chain V-III region B6 | 0.25 (0.00 - 3.44) | 0.33 (0.00 - 1.31) | 0.904 |
| Ig lambda chain V-I region NEW | 0.07 (0.00 - 2.33) | 0.12 (0.00 - 1.36) | 0.554 |
| Ig lambda chain V-I region NEWM | 0.13 (0.00 - 5.67) | 0.14 (0.00 - 1.40) | 0.620 |
| Ig lambda chain V-II region BOH | 0.00 (0.00 - 2.25) | 0.00 (0.00 - 0.00) | 0.431 |
| Ig lambda chain V-IV region Hil | 0.24 (0.00 - 1.32) | 0.15 (0.00 - 1.23) | 0.240 |
| Ig lambda-2 chain C regions | 3.02 (0.60 - 22.08) | 3.17 (1.28 - 7.43) | 0.539 |
| Inter-alpha-trypsin inhibitor heavy chain H4 | 0.20 (0.06 - 0.43) | 0.20 (0.15 - 0.28) | 0.766 |
| Leucine-rich alpha-2-glycoprotein | 0.15 (0.02 - 0.63) | 0.21 (0.12 - 0.34) | 0.018 |
| Pigment epithelium-derived factor | 0.07 (0.02 - 0.18) | 0.06 (0.02 - 0.11) | 0.362 |
| Selenoprotein P | 0.02 (0.00 - 0.06) | 0.02 (0.00 - 0.03) | 0.493 |
| Sialic acid-binding Ig-like lectin 16 | 0.00 (0.00 - 0.03) | 0.01 (0.00 - 0.02) | 0.254 |
| TPR and ankyrin repeat-containing protein 1 | 0.00 (0.00 - 0.00) | 0.00 (0.00 - 0.00) | 0.688 |
| UDP-glucose:glycoprotein glucosyltransferase 2 | 0.00 (0.00 - 0.00) | 0.00 (0.00 - 0.01) | 0.657 |
| von Willebrand factor | 0.01 (0.00 - 0.05) | 0.01 (0.00 - 0.03) | 0.721 |
|  |  |  |  |
| ***Lower in tumor*** |  |  |  |
| 72 kDa inositol polyphosphate 5-phosphatase | 0.00 (0.00 - 0.06) | 0.00 (0.00 - 0.02) | 0.565 |
| Ankyrin repeat and sterile alpha motif domain-containing protein 1B | 0.00 (0.00 - 0.01) | 0.00 (0.00 - 0.01) | 0.669 |
| Apolipoprotein A-I | 8.31 (1.22 - 29.24) | 4.31 (1.83 - 14.73) | 0.113 |
| Biotinidase | 0.00 (0.00 - 0.03) | 0.00 (0.00 - 0.01) | 0.177 |
| Carboxypeptidase B2 | 0.00 (0.00 - 0.02) | 0.01 (0.00 - 0.05) | 0.004 |
| Complement C3 | 0.91 (0.34 - 1.67) | 1.24 (0.79 - 1.82) | 0.004 |
| Cystatin-F | 0.00 (0.00 - 0.67) | 0.00 (0.00 - 0.12) | 0.499 |
| Dynein heavy chain domain-containing protein 1 | 0.00 (0.00 - 0.00) | 0.00 (0.00 - 0.00) | 0.401 |
| Hepatocyte growth factor activator | 0.00 (0.00 - 0.02) | 0.00 (0.00 - 0.02) | 0.516 |
| Ig lambda chain V region 4A | 0.09 (0.00 - 0.86) | 0.11 (0.00 - 0.28) | 0.371 |
| Ig mu chain C region | 0.21 (0.00 - 0.60) | 0.27 (0.00 - 0.35) | 0.689 |
| Insulin-like growth factor-binding protein complex acid labile subunit | 0.01 (0.00 - 0.05) | 0.02 (0.00 - 0.05) | 0.342 |
| Kinesin heavy chain isoform 5C | 0.00 (0.00 - 0.03) | 0.00 (0.00 - 0.03) | 0.537 |
| Kinesin-like protein KIF13B | 0.00 (0.00 - 0.01) | 0.00 (0.00 - 0.01) | 0.954 |
| *N*-acetylmuramoyl-L-alanine amidase | 0.04 (0.00 - 0.09) | 0.03 (0.01 - 0.09) | 0.634 |
| Pericentriolar material 1 protein | 0.00 (0.00 - 0.03) | 0.00 (0.00 - 0.00) | 0.309 |
| Phosphatidylinositol-glycan-specific phospholipase D | 0.00 (0.00 - 0.03) | 0.01 (0.00 - 0.02) | 0.010 |
| Platelet basic protein | 0.04 (0.00 - 0.38) | 0.11 (0.00 - 0.41) | 0.032 |
| Platelet factor 4 | 0.00 (0.00 - 0.04) | 0.04 (0.00 - 0.41) | 0.005 |
| Protein MENT | 0.00 (0.00 - 0.02) | 0.00 (0.00 - 0.03) | 0.148 |
| Prothrombin | 0.23 (0.08 - 0.54) | 0.23 (0.09 - 0.48) | 0.736 |
| Pseudouridylate synthase 7 homolog-like protein | 0.00 (0.00 - 0.05) | 0.00 (0.00 - 0.04) | 0.944 |
| Retinol-binding protein 4 | 0.13 (0.00 - 0.56) | 0.31 (0.08 - 0.74) | 0.013 |
| Serotransferrin | 3.76 (1.05 - 7.76) | 4.82 (2.62 - 7.40) | 0.100 |
| Serum albumin | 33.01 (2.20 - 72.20) | 36.37 (2.45 - 80.78) | 0.488 |
| Serum paraoxonase/arylesterase 1 | 0.08 (0.00 - 0.29) | 0.11 (0.07 - 0.19) | 0.142 |
| Spectrin beta chain, non-erythrocytic 4 | 0.00 (0.00 - 0.01) | 0.00 (0.00 - 0.01) | 0.985 |
| Tetranectin | 0.03 (0.00 - 0.27) | 0.03 (0.00 - 0.13) | 0.629 |
| THAP domain-containing protein 4 | 0.00 (0.00 - 0.12) | 0.00 (0.00 - 0.06) | 0.904 |
| Thrombospondin-1 | 0.00 (0.00 - 0.04) | 0.01 (0.00 - 0.04) | 0.070 |
| Thymosin beta-4 | 0.00 (0.00 - 0.28) | 0.00 (0.00 - 0.11) | 0.869 |
| Trinucleotide repeat-containing gene 6C protein | 0.00 (0.00 - 0.03) | 0.00 (0.00 - 0.02) | 0.866 |
| Vasodilator-stimulated phosphoprotein | 0.00 (0.00 - 0.02) | 0.00 (0.00 - 0.00) | 0.524 |
| Data are median values (minimum - maximum). Variables are compared using Mann-Whitney *U* tests. CCA, cholangiocarcinoma. | | | |
