# Supplementary material for: Plasma proteome plus site‐specific N‐glycoprofiling for hepatobiliary carcinomas
Source: J Pathol Clin Res. 2019 Jun 25;5(3):199–212. doi: 10.1002/cjp2.136 (PMC6648390; doi:10.1002/cjp2.136)
Supplement: Supplementary file 7 — Table S6. Relationship between age and differential protein content in patients with hepatobiliary cancers [file CJP2-5-199-s007.docx]

**Plasma proteome plus site-specific *N*-glycoprofiling for hepatobiliary carcinomas**

Chang T-T *et al*. *J Pathol Clin Res* DOI: 10.1002/cjp2.136

| **Table S6.** Relationship between age and differential protein content in patients with hepatobiliary cancers (n = 220) | | |
| --- | --- | --- |
| Variable | Coefficient *r* | *P*-value |
| ***Higher in tumor*** |  |  |
| 2-hydroxyacylsphingosine 1-beta-galactosyltransferase | -0.038 | 0.578 |
| Apolipoprotein C-III | -0.072 | 0.285 |
| BPI fold-containing family C protein | -0.095 | 0.162 |
| Carbonic anhydrase 1 | 0.112 | 0.098 |
| Coagulation factor XIII A chain | 0.000 | 0.989 |
| C-reactive protein | 0.005 | 0.943 |
| Galectin-3-binding protein | 0.130 | 0.055 |
| Ig heavy chain V-III region KOL | -0.016 | 0.811 |
| Ig heavy chain V-III region NIE | -0.010 | 0.879 |
| Ig kappa chain C region | -0.014 | 0.838 |
| Ig kappa chain V-III region B6 | -0.030 | 0.661 |
| Ig lambda chain V-I region NEW | -0.043 | 0.528 |
| Ig lambda chain V-I region NEWM | 0.038 | 0.576 |
| Ig lambda chain V-II region BOH | -0.067 | 0.324 |
| Ig lambda chain V-IV region Hil | -0.072 | 0.288 |
| Ig lambda-2 chain C regions | -0.007 | 0.919 |
| Inter-alpha-trypsin inhibitor heavy chain H4 | -0.034 | 0.619 |
| Leucine-rich alpha-2-glycoprotein | -0.086 | 0.204 |
| Pigment epithelium-derived factor | -0.058 | 0.395 |
| Selenoprotein P | -0.044 | 0.513 |
| Sialic acid-binding Ig-like lectin 16 | 0.033 | 0.621 |
| TPR and ankyrin repeat-containing protein 1 | -0.019 | 0.782 |
| UDP-glucose:glycoprotein glucosyltransferase 2 | -0.095 | 0.160 |
| von Willebrand factor | 0.039 | 0.569 |
|  |  |  |
| ***Lower in tumor*** |  |  |
| 72 kDa inositol polyphosphate 5-phosphatase | -0.021 | 0.756 |
| Ankyrin repeat and sterile alpha motif domain-containing protein 1B | 0.019 | 0.780 |
| Apolipoprotein A-I | -0.110 | 0.105 |
| Biotinidase | -0.101 | 0.134 |
| Carboxypeptidase B2 | 0.008 | 0.901 |
| Complement C3 | -0.021 | 0.762 |
| Cystatin-F | 0.044 | 0.514 |
| Dynein heavy chain domain-containing protein 1 | 0.015 | 0.821 |
| Hepatocyte growth factor activator | -0.118 | 0.081 |
| Ig lambda chain V region 4A | 0.054 | 0.425 |
| Ig mu chain C region | 0.130 | 0.054 |
| Insulin-like growth factor-binding protein complex acid labile subunit | -0.284 | <0.001 |
| Kinesin heavy chain isoform 5C | 0.001 | 0.987 |
| Kinesin-like protein KIF13B | 0.007 | 0.914 |
| *N*-acetylmuramoyl-L-alanine amidase | -0.040 | 0.557 |
| Pericentriolar material 1 protein | 0.057 | 0.401 |
| Phosphatidylinositol-glycan-specific phospholipase D | -0.178 | 0.008 |
| Platelet basic protein | -0.116 | 0.087 |
| Platelet factor 4 | -0.019 | 0.776 |
| Protein MENT | -0.034 | 0.619 |
| Prothrombin | -0.048 | 0.476 |
| Pseudouridylate synthase 7 homolog-like protein | 0.067 | 0.323 |
| Retinol-binding protein 4 | -0.021 | 0.759 |
| Serotransferrin | -0.070 | 0.299 |
| Serum albumin | -0.016 | 0.815 |
| Serum paraoxonase/arylesterase 1 | -0.088 | 0.192 |
| Spectrin beta chain, non-erythrocytic 4 | 0.084 | 0.212 |
| Tetranectin | -0.039 | 0.569 |
| THAP domain-containing protein 4 | 0.012 | 0.865 |
| Thrombospondin-1 | -0.120 | 0.077 |
| Thymosin beta-4 | 0.015 | 0.823 |
| Trinucleotide repeat-containing gene 6C protein | 0.036 | 0.596 |
| Vasodilator-stimulated phosphoprotein | 0.041 | 0.542 |
| Results were obtained from Pearson correlation tests. | | |
