# Supplementary material for: Plasma proteome plus site‐specific N‐glycoprofiling for hepatobiliary carcinomas
Source: J Pathol Clin Res. 2019 Jun 25;5(3):199–212. doi: 10.1002/cjp2.136 (PMC6648390; doi:10.1002/cjp2.136)
Supplement: Supplementary file 8 — Table S7. Differential protein expression in patients with hepatocellular carcinoma with different risk factors [file CJP2-5-199-s008.docx]

**Plasma proteome plus site-specific *N*-glycoprofiling for hepatobiliary carcinomas**

Chang T-T *et al*. *J Pathol Clin Res* DOI: 10.1002/cjp2.136

| **Table S7.** Differential protein expression in patients with hepatocellular carcinoma (n = 148) with different risk factors | | | |
| --- | --- | --- | --- |
| Hepatitis B (n = 84) | Hepatitis C (n = 49) | Cirrhosis (n = 90) | Steatosis (n = 34) |
| ***Upregulated*** |  |  |  |
| Phosphatidylinositol-glycan-specific phospholipase D | Serotransferrin | Galetin-3-binding protein^†^ | 2-hydroxyacylsphingosine 1-beta-galactosyltransferase^‡^ |
| Selenoprotein P |  | Tetranectin | Apolipoprotein C-III |
|  |  |  | Complement C3 |
|  |  |  | Galectin-3-binding protein |
|  |  |  | Inter-alpha-trypsin inhibitor heavy chain H4 |
|  |  |  | Leucine-rich alpha-2-glycoprotein |
|  |  |  | Prothrombin^†^ |
|  |  |  | Sialic acid-binding Ig-like lectin 16^‡^ |
|  |  |  | TPR and ankyrin repeat-containing protein 1^†^ |
| ***Downregulated*** |  |  |  |
| Serotransferrin | Apolipoprotein A-I | Platelet basic protein | Biotinidase^†^ |
|  | Coagulation factor XIII A chain | Prothrombin | Phosphatidylinositol-glycan-specific phospholipase D^†^ |
|  | Insulin-like growth factor-binding protein complex acid labile subunit^†^ | Serum albumin |  |
|  | Serum paraoxonase/arylesterase 1^†^ |  |  |
| Protein content [molecular %; exponentially modified protein abundance index (emPAI)/Σ(emPAI) × 100] was used for the protein semi-quantification. *N*-glycoproteins are shown in underlines.  Data are analyzed using Mann-Whitney U tests. *P*-values are < 0.05 except those are marked with ^†^(P < 0.01) and ^‡^(P< 0.001). | | | |
