# Supplementary material for: Plasma proteome plus site‐specific N‐glycoprofiling for hepatobiliary carcinomas
Source: J Pathol Clin Res. 2019 Jun 25;5(3):199–212. doi: 10.1002/cjp2.136 (PMC6648390; doi:10.1002/cjp2.136)
Supplement: Supplementary file 9 — Table S8. Complement C3 asparagine85 glycoprofiles [file CJP2-5-199-s009.docx]

**Plasma proteome plus site-specific *N*-glycoprofiling for hepatobiliary carcinomas**

Chang T-T *et al*. *J Pathol Clin Res* DOI: 10.1002/cjp2.136

| **Table S8.** Complement C3 asparagine85 glycoprofiles | | | | | | |
| --- | --- | --- | --- | --- | --- | --- |
| Glycoform | m/z | Control | HCC | CCA | cHCC-CCA | *P*-value |
| Hex5HexNAc2 (Man5), % | 1157.8661 (3+) | 16.4 (8.4 – 28.9) | 13.9 (0.0 – 29.4) | 12.5 (3.6 – 22.6) | 12.9 (7.9 – 19.7) | 0.025 |
| Hex6HexNAc2 (Man6), % | 1211.8837 (3+) | 76.1 (59.9 – 80.8) | 75.8 (52.4 – 100.0) | 76.9 (68.5 – 93.3) | 77.7 (69.4 – 85.5) | 0.103 |
| Hex7HexNAc2 (Man7), % | 1265.9013 (3+) | 7.8 (2.4 – 18.7) | 9.8 (0.0 – 30.5) | 7.9 (0.0 – 22.3) | 8.9 (5.5 – 14.3) | 0.030 |
| Hex8HexNAc2 (Man8), % | 1319.9189 (3+) | 0.0 (0.0 – 0.3) | 0.0 (0.0 – 0.6) | 0.0 (0.0 – 1.1) | 0.0 (0.0 – 0.0) | 0.340 |
| Hex6HexNAc3SA1 (Hybrid), % | 1376.6087 (3+) | 0.1 (0.0 – 1.1) | 0.0 (0.0 – 0.6) | 0.0 (0.0 – 1.3) | 0.0 (0.0 – 0.6) | 0.002 |
| Data are median values (minimum - maximum). *P*-values are obtained from Kruskal-Wallis tests.  Abbreviations: CCA, cholangiocarcinoma; cHCC-CCA, combined hepatocellular carcinoma and cholangiocarcinoma; HCC, hepatocellular carcinoma; Hex, hexose; HexNAc, *N*-acetylhexosamine; man, mannosylation; m/z, mass-to-charge ratio; SA, sialic acid | | | | | | |
