# Supplementary material for: Plasma proteome plus site‐specific N‐glycoprofiling for hepatobiliary carcinomas
Source: J Pathol Clin Res. 2019 Jun 25;5(3):199–212. doi: 10.1002/cjp2.136 (PMC6648390; doi:10.1002/cjp2.136)
Supplement: Supplementary file 10 — Table S9. Complement C3 Asparagine85 glycoprofiles in patients with hepatocellular carcinoma [file CJP2-5-199-s010.docx]

**Plasma proteome plus site-specific *N*-glycoprofiling for hepatobiliary carcinomas**

Chang T-T *et al*. *J Pathol Clin Res* DOI: 10.1002/cjp2.136

| **Table S9.** Complement C3 Asparagine85 glycoprofiles in patients with hepatocellular carcinoma | | | | | |
| --- | --- | --- | --- | --- | --- |
| Number | Hex5HexNAc2 (Man5), % | Hex6HexNAc2 (Man6), % | Hex7HexNAc2 (Man7), % | Hex8HexNAc2 (Man8), % | Hex6HexNAc3SA1 (Hybrid), % |
| 1 | 15.02 | 75.48 | 9.50 | 0.00 | 0.00 |
| 2 | 10.85 | 79.51 | 9.64 | 0.00 | 0.00 |
| 3 | 15.39 | 78.22 | 6.38 | 0.00 | 0.00 |
| 4 | 23.68 | 57.44 | 18.26 | 0.14 | 0.48 |
| 5 | 12.20 | 76.80 | 10.62 | 0.00 | 0.38 |
| 6 | 5.46 | 65.75 | 28.23 | 0.55 | 0.00 |
| 7 | 10.69 | 69.22 | 19.69 | 0.40 | 0.00 |
| 8 | 9.83 | 76.79 | 13.39 | 0.00 | 0.00 |
| 9 | 16.96 | 73.37 | 9.67 | 0.00 | 0.00 |
| 10 | 12.47 | 72.12 | 15.41 | 0.00 | 0.00 |
| 11 | 16.57 | 75.96 | 7.47 | 0.00 | 0.00 |
| 12 | 13.49 | 80.09 | 6.42 | 0.00 | 0.00 |
| 13 | 16.59 | 77.54 | 5.87 | 0.00 | 0.00 |
| 14 | 10.01 | 70.93 | 19.06 | 0.00 | 0.00 |
| 15 | 15.20 | 73.42 | 11.33 | 0.00 | 0.06 |
| 16 | 20.70 | 75.08 | 4.22 | 0.00 | 0.00 |
| 17 | 11.43 | 78.65 | 9.64 | 0.29 | 0.00 |
| 18 | 13.78 | 74.76 | 11.45 | 0.00 | 0.00 |
| 19 | 16.20 | 73.99 | 9.81 | 0.00 | 0.00 |
| 20 | 10.08 | 79.48 | 10.44 | 0.00 | 0.00 |
| 21 | 16.20 | 73.99 | 9.81 | 0.00 | 0.00 |
| 22 | 13.29 | 73.81 | 12.91 | 0.00 | 0.00 |
| 23 | 16.66 | 78.83 | 4.51 | 0.00 | 0.00 |
| 24 | 0.00 | 75.53 | 24.47 | 0.00 | 0.00 |
| 25 | 13.46 | 76.36 | 9.81 | 0.00 | 0.37 |
| 26 | 14.86 | 72.78 | 12.35 | 0.00 | 0.00 |
| 27 | 24.39 | 71.48 | 4.14 | 0.00 | 0.00 |
| 28 | 10.53 | 71.49 | 17.76 | 0.23 | 0.00 |
| 29 | 9.11 | 75.83 | 15.06 | 0.00 | 0.00 |
| 30 | 13.95 | 74.95 | 11.10 | 0.00 | 0.00 |
| 31 | 14.15 | 74.56 | 11.29 | 0.00 | 0.00 |
| 32 | 14.10 | 76.03 | 9.87 | 0.00 | 0.00 |
| 33 | 10.25 | 77.37 | 12.39 | 0.00 | 0.00 |
| 34 | 9.22 | 82.44 | 8.33 | 0.00 | 0.00 |
| 35 | 10.93 | 72.49 | 15.93 | 0.65 | 0.00 |
| 36 | 13.34 | 72.61 | 14.05 | 0.00 | 0.00 |
| 37 | 29.38 | 61.57 | 8.86 | 0.02 | 0.16 |
| 38 | 9.82 | 76.92 | 13.26 | 0.00 | 0.00 |
| 39 | 10.04 | 75.77 | 14.18 | 0.00 | 0.00 |
| 40 | 11.84 | 72.66 | 15.15 | 0.34 | 0.00 |
| 41 | 13.91 | 78.56 | 7.49 | 0.04 | 0.00 |
| 42 | 10.80 | 73.63 | 15.57 | 0.00 | 0.00 |
| 43 | 10.80 | 73.63 | 15.57 | 0.00 | 0.00 |
| 44 | 9.61 | 71.00 | 19.39 | 0.00 | 0.00 |
| 45 | 10.83 | 73.80 | 15.36 | 0.00 | 0.00 |
| 46 | 11.42 | 80.40 | 8.18 | 0.00 | 0.00 |
| 47 | 17.55 | 74.06 | 8.39 | 0.00 | 0.00 |
| 48 | 14.26 | 76.81 | 8.93 | 0.00 | 0.00 |
| 49 | 13.66 | 76.94 | 9.40 | 0.00 | 0.00 |
| 50 | 15.32 | 77.34 | 7.34 | 0.00 | 0.00 |
| 51 | 12.17 | 80.30 | 7.53 | 0.00 | 0.00 |
| 52 | 17.01 | 72.79 | 10.20 | 0.00 | 0.00 |
| 53 | 10.24 | 82.97 | 6.79 | 0.00 | 0.00 |
| 54 | 14.35 | 70.29 | 14.77 | 0.00 | 0.59 |
| 55 | 15.16 | 74.44 | 10.22 | 0.00 | 0.17 |
| 56 | 15.25 | 74.77 | 9.98 | 0.00 | 0.00 |
| 57 | 19.42 | 74.89 | 5.69 | 0.00 | 0.00 |
| 58 | 13.60 | 79.31 | 7.09 | 0.00 | 0.00 |
| 59 | 15.18 | 74.91 | 9.87 | 0.00 | 0.05 |
| 60 | 19.77 | 71.68 | 8.12 | 0.00 | 0.43 |
| 61 | 13.59 | 73.20 | 13.17 | 0.00 | 0.04 |
| 62 | 10.22 | 74.59 | 15.19 | 0.00 | 0.00 |
| 63 | 12.62 | 72.62 | 14.76 | 0.00 | 0.00 |
| 64 | 12.57 | 71.67 | 15.76 | 0.00 | 0.00 |
| 65 | 13.01 | 68.80 | 18.19 | 0.00 | 0.00 |
| 66 | 13.46 | 76.38 | 10.15 | 0.00 | 0.00 |
| 67 | 16.29 | 72.88 | 10.83 | 0.00 | 0.00 |
| 68 | 8.80 | 75.15 | 16.05 | 0.00 | 0.00 |
| 69 | 13.48 | 71.49 | 15.03 | 0.00 | 0.00 |
| 70 | 15.72 | 75.96 | 8.20 | 0.00 | 0.12 |
| 71 | 11.54 | 76.75 | 11.71 | 0.00 | 0.00 |
| 72 | 15.96 | 77.19 | 6.85 | 0.00 | 0.00 |
| 73 | 8.41 | 79.04 | 12.56 | 0.00 | 0.00 |
| 74 | 14.19 | 74.16 | 11.65 | 0.00 | 0.00 |
| 75 | 13.11 | 77.45 | 9.45 | 0.00 | 0.00 |
| 76 | 10.01 | 75.74 | 14.25 | 0.00 | 0.00 |
| 77 | 9.05 | 81.08 | 9.87 | 0.00 | 0.00 |
| 78 | 16.51 | 72.86 | 10.63 | 0.00 | 0.00 |
| 79 | 20.99 | 72.52 | 6.48 | 0.00 | 0.00 |
| 80 | 16.95 | 76.92 | 6.13 | 0.00 | 0.00 |
| 81 | 15.66 | 76.55 | 7.78 | 0.00 | 0.00 |
| 82 | 21.29 | 78.71 | 0.00 | 0.00 | 0.00 |
| 83 | 8.72 | 72.21 | 18.98 | 0.09 | 0.00 |
| 84 | 15.67 | 75.07 | 9.25 | 0.00 | 0.00 |
| 85 | 12.57 | 77.46 | 9.97 | 0.00 | 0.00 |
| 86 | 14.92 | 74.88 | 10.20 | 0.00 | 0.00 |
| 87 | 18.46 | 74.90 | 6.64 | 0.00 | 0.00 |
| 88 | 17.85 | 75.19 | 6.46 | 0.00 | 0.50 |
| 89 | 20.42 | 79.56 | 0.02 | 0.00 | 0.00 |
| 90 | 17.03 | 52.45 | 30.52 | 0.00 | 0.00 |
| 91 | 16.31 | 73.91 | 9.78 | 0.00 | 0.00 |
| 92 | 0.00 | 100.00 | 0.00 | 0.00 | 0.00 |
| 93 | 0.00 | 100.00 | 0.00 | 0.00 | 0.00 |
| 94 | 0.00 | 100.00 | 0.00 | 0.00 | 0.00 |
| 95 | 1.73 | 82.95 | 15.32 | 0.00 | 0.00 |
| 96 | 0.00 | 79.73 | 20.27 | 0.00 | 0.00 |
| 97 | 10.86 | 68.95 | 20.19 | 0.00 | 0.00 |
| 98 | 10.62 | 73.58 | 15.64 | 0.10 | 0.06 |
| 99 | 0.00 | 100.00 | 0.00 | 0.00 | 0.00 |
| 100 | 4.66 | 84.83 | 10.50 | 0.00 | 0.00 |
| 101 | 11.13 | 73.30 | 15.25 | 0.00 | 0.32 |
| 102 | 15.37 | 76.70 | 7.93 | 0.00 | 0.00 |
| 103 | 18.38 | 77.10 | 4.52 | 0.00 | 0.00 |
| 104 | 9.20 | 75.02 | 15.79 | 0.00 | 0.00 |
| 105 | 19.94 | 74.24 | 5.81 | 0.00 | 0.00 |
| 106 | 11.92 | 77.25 | 10.84 | 0.00 | 0.00 |
| 107 | 16.37 | 74.76 | 8.77 | 0.00 | 0.10 |
| 108 | 11.80 | 79.16 | 9.04 | 0.00 | 0.00 |
| 109 | 10.99 | 82.44 | 6.56 | 0.00 | 0.00 |
| 110 | 8.13 | 67.07 | 24.80 | 0.00 | 0.00 |
| 111 | 13.23 | 76.43 | 10.27 | 0.00 | 0.06 |
| 112 | 18.78 | 70.07 | 10.91 | 0.00 | 0.24 |
| 113 | 18.99 | 75.76 | 5.25 | 0.00 | 0.00 |
| 114 | 29.44 | 70.29 | 0.00 | 0.00 | 0.27 |
| 115 | 18.87 | 72.47 | 8.64 | 0.00 | 0.02 |
| 116 | 16.34 | 76.96 | 6.70 | 0.00 | 0.00 |
| 117 | 16.54 | 75.66 | 7.81 | 0.00 | 0.00 |
| 118 | 14.89 | 78.30 | 6.76 | 0.00 | 0.06 |
| 119 | 10.70 | 77.71 | 11.60 | 0.00 | 0.00 |
| 120 | 13.71 | 77.09 | 9.02 | 0.15 | 0.03 |
| 121 | 18.07 | 76.91 | 5.00 | 0.00 | 0.02 |
| 122 | 16.50 | 78.12 | 5.39 | 0.00 | 0.00 |
| 123 | 14.72 | 78.84 | 6.44 | 0.00 | 0.00 |
| 124 | 18.11 | 73.15 | 8.61 | 0.00 | 0.13 |
| 125 | 11.17 | 78.56 | 10.25 | 0.00 | 0.02 |
| 126 | 15.00 | 80.48 | 4.43 | 0.00 | 0.08 |
| 127 | 14.97 | 78.93 | 5.90 | 0.00 | 0.20 |
| 128 | 15.03 | 73.68 | 11.28 | 0.00 | 0.02 |
| 129 | 16.71 | 74.38 | 8.85 | 0.00 | 0.06 |
| 130 | 13.42 | 78.55 | 8.03 | 0.00 | 0.00 |
| 131 | 19.41 | 75.25 | 5.28 | 0.00 | 0.06 |
| 132 | 15.30 | 74.80 | 9.90 | 0.00 | 0.00 |
| 133 | 20.22 | 74.21 | 5.42 | 0.00 | 0.15 |
| 134 | 12.02 | 78.00 | 9.52 | 0.00 | 0.46 |
| 135 | 15.51 | 76.56 | 7.88 | 0.00 | 0.05 |
| 136 | 18.32 | 73.38 | 8.25 | 0.00 | 0.06 |
| 137 | 11.08 | 78.20 | 10.56 | 0.00 | 0.17 |
| 138 | 13.82 | 73.68 | 12.31 | 0.03 | 0.15 |
| 139 | 13.50 | 77.35 | 9.08 | 0.00 | 0.08 |
| 140 | 14.80 | 78.18 | 7.02 | 0.00 | 0.00 |
| 141 | 17.78 | 76.58 | 5.64 | 0.00 | 0.00 |
| 142 | 15.67 | 80.48 | 3.35 | 0.00 | 0.50 |
| 143 | 15.70 | 78.11 | 6.04 | 0.00 | 0.14 |
| 144 | 12.20 | 79.07 | 8.55 | 0.04 | 0.14 |
| 145 | 14.27 | 76.09 | 9.27 | 0.00 | 0.37 |
| 146 | 16.80 | 76.09 | 6.83 | 0.00 | 0.28 |
| 147 | 12.53 | 77.34 | 10.07 | 0.00 | 0.06 |
| 148 | 12.09 | 76.25 | 11.66 | 0.00 | 0.00 |
| Data are percentages.  Abbreviations: Hex, hexose; HexNAc, *N*-acetylhexosamine; man, mannosylation; SA, sialic acid | | | | | |
