# Supplementary material for: Plasma proteome plus site‐specific N‐glycoprofiling for hepatobiliary carcinomas
Source: J Pathol Clin Res. 2019 Jun 25;5(3):199–212. doi: 10.1002/cjp2.136 (PMC6648390; doi:10.1002/cjp2.136)
Supplement: Supplementary file 11 — Table S10. Relationship between age and complement C3 asparagine85 glycoprofile in patients with hepatocellular carcinoma [file CJP2-5-199-s011.docx]

**Plasma proteome plus site-specific *N*-glycoprofiling for hepatobiliary carcinomas**

Chang T-T *et al*. *J Pathol Clin Res* DOI: 10.1002/cjp2.136

| **Table S10.** Relationship between age and complement C3 asparagine85 glycoprofile in patients with hepatocellular carcinoma (n = 148) | | |
| --- | --- | --- |
| Variable | Coefficient *r* | *P*-value |
| Hex5HexNAc2 (Man5), % | -0.045 | 0.585 |
| Hex6HexNAc2 (Man6), % | 0.082 | 0.322 |
| Hex7HexNAc2 (Man7), % | -0.044 | 0.592 |
| Hex8HexNAc2 (Man8), % | -0.088 | 0.287 |
| Hex6HexNAc3SA1 (Hybrid), % | -0.127 | 0.123 |
| Hex5HexNAc2 (Man5), g/L | -0.069 | 0.405 |
| Hex6HexNAc2 (Man6), g/L | -0.047 | 0.570 |
| Hex7HexNAc2 (Man7), g/L | -0.076 | 0.359 |
| Hex8HexNAc2 (Man8), g/L | -0.090 | 0.274 |
| Hex6HexNAc3SA1 (Hybrid), g/L | -0.098 | 0.238 |
| Results are obtained from Pearson correlation tests. Abbreviations: Hex, hexose; HexNAc, *N*-acetylhexosamine; man, mannosylation; SA, sialic acid. | | |
